# Evolution of the recombination regulator PRDM9 in minke whales

**DOI:** 10.1186/s12864-022-08305-1

**Published:** 2022-03-16

**Authors:** Elena Damm, Kristian K. Ullrich, William B. Amos, Linda Odenthal-Hesse

**Affiliations:** 1 Department Evolutionary Genetics, Research Group Meiotic Recombination and Genome Instability, Max Planck Institute for Evolutionary Biology, August-Thienemann Str. 2, D-24306 Plön, Germany; 2 Department of Zoology, University of Cambridge, Cambridge, UK

**Keywords:** PRDM9, Minke whales, *Balaenoptera acutorostrata*, *Balaenoptera bonaerensis*, Microsatellite loci, mtDNA, Postzygotic reproductive isolation, Meiotic recombination regulation

## Abstract

**Background:**

PRDM9 is a key regulator of meiotic recombination in most metazoans, responsible for reshuffling parental genomes. During meiosis, the PRDM9 protein recognizes and binds specific target motifs via its array of C_2_H_2_ zinc-fingers encoded by a rapidly evolving minisatellite. The gene coding for PRDM9 is the only speciation gene identified in vertebrates to date and shows high variation, particularly in the DNA-recognizing positions of the zinc-finger array, within and between species. Across all vertebrate genomes studied for PRDM9 evolution, only one genome lacks variability between repeat types – that of the North Pacific minke whale. This study aims to understand the evolution and diversity of *Prdm9* in minke whales, which display the most unusual genome reference allele of *Prdm9* so far discovered in mammals.

**Results:**

Minke whales possess all the features characteristic of PRDM9-directed recombination, including complete KRAB, SSXRD and SET domains and a rapidly evolving array of C_2_H_2_-type-Zincfingers (ZnF) with evidence of rapid evolution, particularly at DNA-recognizing positions that evolve under positive diversifying selection. Seventeen novel PRDM9 variants were identified within the Antarctic minke whale species, plus a single distinct PRDM9 variant in Common minke whales – shared across North Atlantic and North Pacific minke whale subspecies boundaries.

**Conclusion:**

The PRDM9 ZnF array evolves rapidly, in minke whales, with at least one DNA-recognizing position under positive selection. Extensive PRDM9 diversity is observed, particularly in the Antarctic in minke whales. Common minke whales shared a specific *Prdm9* allele across subspecies boundaries, suggesting incomplete speciation by the mechanisms associated with PRDM9 hybrid sterility.

**Supplementary Information:**

The online version contains supplementary material available at 10.1186/s12864-022-08305-1.

## Background

The gene *Prdm9* encodes “PR-domain-containing 9” (PRDM9), a meiosis-specific four-domain protein that regulates meiotic recombination in mammalian genomes. The four functional domains of the PRDM9 protein are essential for double-stranded DNA breaks (DSBs) being placed at sequence-specific target sites. Three of the domains are highly conserved: *i)* the N-terminal Kruppel-associated box-domain (KRAB) that promotes protein-protein binding, for example, with EWSR, CXXC1, CDYL and EHMT2 [1, 2]; *ii)* the SSX-repression-domain (SSXRD) of yet unknown function; *iii)* the PR/SET domain, a subclass of the SET domain, with methyltransferase activity at H3K4me3 and H3K36e3. The fourth, C-terminal domain comprises an array of type Cystin_2_Histidin_2_ zinc-fingers (ZnFs), encoded by a minisatellite-like sequence of 84 base pair (bp) tandem repeats. This coding minisatellite reveals evidence of positive selection and concerted evolution, with many functional variants having been found in humans [3, 4], mice [5, 6], non-human primates [7] and other mammals [8, 9]. Even highly domesticated species like equids [8], bovids [10] and ruminants [9, 11] show high diversity and rapid evolution, with considerable variability between minisatellite-like repeat units. In light of the extreme variability between minisatellite-like repeat units in most other vertebrates, one mammal stood out because of its lack of variability - the North Pacific minke whale (*Balaenoptera acutorostrata scammoni*).

Minke whales are marine mammals of the genus *Balaenoptera*, in the parvorder of baleen whales (*Mysticetes*), that are of particular interest not only because little is known about their population biology, seasonal migration routes and breeding behavior but also to support future conservation efforts. Minke whales were long considered a single species but are now classified as two distinct species, the Common minke whale (*Balaenoptera acutorostrata)* and the Antarctic minke whale (*Balaenoptera bonaerensis* Burmeister, 1867). The Common minke whale (*B. acutorostrata)* is cosmopolitan in the waters of the Northern Hemisphere. This species can be separated into two subspecies, the Atlantic minke whale *(B. acutorostrata acutorostrata)* and the North Pacific minke whale (*B. acutorostrata scammoni*), separated from each other by landmasses and the polar ice cap*.* Antarctic minke whale*s* inhabit the waters of the Antarctic ocean in the Southern Hemisphere during feeding season but seasonally migrate to the temperate waters near the Equator during the breeding season [12]. Antarctic minke whale body condition has declined, particularly during the 1990s [13], and anthropogenic pressures such as commercial whaling and future climate change are expected to exacerbate the decline of baleen whales [14].

Although overlapping habitats exist near the Equator, seasonal differences in migration and breeding behavior essentially prevent inter-breeding between Common and Antarctic minke whales [15]. Despite this, occasional migration across the Equator has been observed [16]. Recent studies have uncovered two instances of viable and fertile hybrid individuals, both females and one with a calf most likely sired by an Antarctic minke whale [17, 18]. However, it is unclear whether occasional hybridization events have always occurred or whether they are a recent phenomenon driven by anthropogenic changes, including climate change [17]. More importantly, since both hybrids were females, current data does not exclude postzygotic reproductive isolation mechanisms acting between these species. According to Haldane’s rule, the heterogametic sex would usually become sterile first, which is the male sex in mammals, including minke whales. Hybrid sterility is a universal phenomenon observed in many eukaryotic inter-species hybrids, including yeast, plants, insects, birds, and mammals [19, 20]. Within mammals, it is well characterized how PRDM9 variation between subspecies of mice results in reproductive isolation [21]. In hybrid mice of two different subspecies, variation in PRDM9 ZnF domains leads to asymmetric sets of DSBs in evolutionary divergent homologous genomic sequences [21–23]. This asymmetry likely results from erosion of PRDM9 binding sites via biased gene conversion over long evolutionary timescales [3, 4, 22, 24–29]. As a result, in hybrid genomes, the variant of one species preferentially binds the ancestral binding sites on the homologue of the other species that have not been eroded, and vice versa [21]. The resulting asymmetry of recombination initiation sites is believed to be responsible for the inefficient DSB repair, defective pairing, and asynapsis of the chromosomes in intersubspecific mouse hybrids [21, 30, 31].

Inter-individual *Prdm9* variation has been little studied outside of humans, mice and some domesticated species in which evolutionary constraints may have been relaxed, and little is known about *Prdm9* evolution in non-model organisms. The apparent lack of diversity between minisatellite repeat types coding for the ZnF array in minke whales [32] also offers an unusual opportunity to study *Prdm9* as the only known mammalian ‘speciation’ locus. In light of recent reports of interspecies hybrids, secondary admixture and aberrant migration patterns, due to global warming, this is especially interesting.

## Results

### The evolutionary context of PRDM9 in *Artiodactyla*

**Fig. 1:**
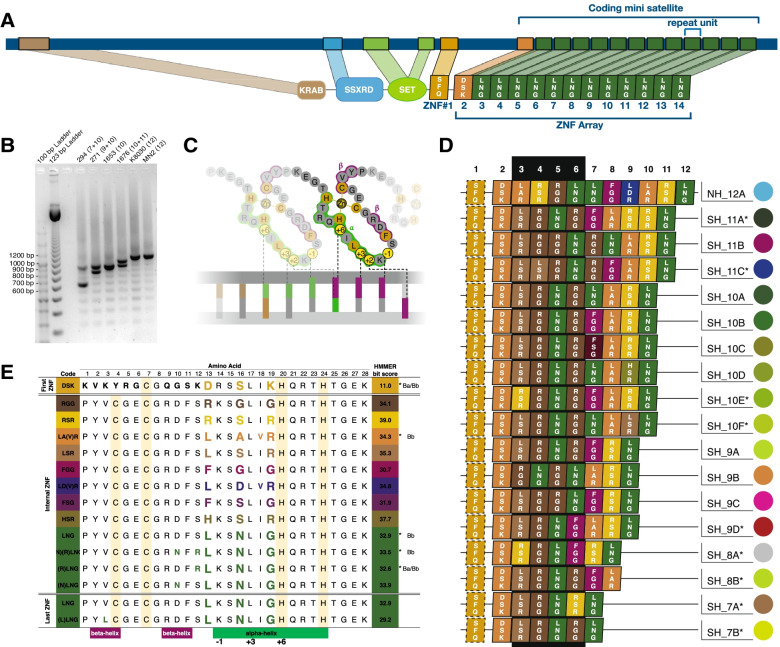
Diversity in the Cys2His2-ZnF domain in minke whales. **A** *Prdm9* gene annotation in *Balaenoptera acutorostrata scammoni* genome reference**,** with primer site annotations and predicted PRDM9 protein from nucleotide translation (**B**) Representative PCR products of DNAs from individuals from AN IV (294,271), AN V (1653), NP (K8030) and NA (MN2), showing variation in the number of minisatellite repeats units (number of ZnFs in brackets) (**C**) Stylized structure of ZnF binding to DNA, with nucleotide-specificity conferred by amino-acids in positions − 1, + 2, + 3, and + 6 of alpha-helices. **D** PRDM9 ZnF arrays identified in minke whales, named with broad-scale sampling location, Northern Hemisphere (NH) and Southern Hemisphere (SH) and the total number of ZnFs in the ZnFs domain. **E** Types of PRDM9 ZnFs in minke whales. Three-letter codes were generated using the IUPAC nomenclature of amino-acids involved in DNA binding. All variable amino acids are colored, and asterisks label ZnFs also present in genome references of *Balaenoptera acutorostrata scammoni* (*Ba) or *Balaenoptera bonaerensis* (*Bb), see also Additional File 6

Full-length PRDM9 orthologues had previously been identified in common minke whale *Balaenoptera acutorostrata scammoni* and the bottlenose dolphin (*Tursiops truncatus*) [32]. For a broad view on the evolution of PRDM9 in even-toed ungulates (Artiodactyla), the *Balaenoptera acutorostrata scammoni* protein (Fig. 1A) was used as a query to search for PRDM9 orthologs in all Artiodactyla, where genomic resources were publicly available (Additional File 1). Complete proximal PRDM9 domains comprising KRAB, SSXRD and SET were also identified in Antarctic minke whales *Balaenoptera bonaerensis*) and all other Artiodactyla in our dataset (Additional File 1 and Additional File 2). Phylogenetic analyses on concatenated protein-coding amino-acid sequences of the N-terminal domains established an evolutionary context of PRDM9 orthologues, as shown in Additional File 3. This phylogenic tree separates Suidae and Ruminantia from Whippomorpha, which split into one branch leading to Hippopotamus and another to Cetacea. Within Cetacea, two distinct branches divide Odontocetes (toothed whales) and Mysticetes (baleen whales), which include the minke whale (Additional File 3). We extended the analyses across Artiodactyla*,* and found the ZnF domain was present in all available Cetartiodactyla genomes*,* except Hippopotamus. However the number of ZnF that could be recovered varied across species (Additional File 3). The complete PRDM9 ZnF domain comprises an array of ZnFs (the ZnF-array), as well as a single zinc-knuckle that is located proximally. Within each ZnF, the DNA-contacting residues (position 13, 16 and 19) of the alpha helix are responsible for DNA-binding (as depicted in Fig. 1C). The zinc knuckle possesses the same DNA contacting residues Serine, Phenylalanine and Glutamine (“SFQ”) in all Mysticetes, Ruminantia and Suidae. In Odontocetes, phenylalanine at position 16 is replaced with Isoleucine, resulting in DNA-contacting amino-acid residues “SIQ” (Additional File 4).

The first ZnF at the start of the ZnF-array is identical between the available Common minke whale genome (belonging to the subspecies *Balaenoptera acutorostrata scammoni*) and the Antarctic minke whale (*Balaenoptera bonaerensis*) as well as the blue whale (*Balaenoptera musculus*) (Additional File 5). Only a single amino-acid change at position 22 is seen between these *Balaenoptera* and the bowhead whale (*Balaena mysticetus*). However, the DNA contacting residues Aspartic Acid, Serine and Lysine “DSK” are identical across Cetacea, with Ruminantia differing by a single DNA-binding amino-acid change from Lysine to Threonine at position 19. Additional changes are seen at amino acids not responsible for DNA binding specificity, including 5, 6, 18, and 24. Here amino acid position 18 is identical in Mysticetes and Ruminantia but distinguishes Odontocetes, and position six distinguishes Delphinidae and Ruminantia from other Artiodactyla (Additional File 5).

### Characterizing the *Prdm9* gene in minke whales

To characterize the sequence and structure of the *Prdm9* gene in minke whales beyond the genome reference sequences, long-range phased sequencing was applied. Under the assumption that *Prdm9* would display little to no variability between repeat types, *Prdm9* was amplified and sequenced from a pooled sample, containing DNA from six individuals, five Antarctic minke whales and one common minke whale (reflecting the ratio of available Antarctic minke whale and common minke whale samples). The consensus sequence was subjected to *in-silico* prediction that successfully recovered all relevant PRDM9 protein domains with high-confidence: the KRAB domain (E-value: 1.84e^− 11^); the SSXRD motif (E-value: 4.46e^− 10^); the PR/SET domain (E-value: 9.69e^− 05^); and several Zinc-Fingers (Fig. 1A), including a proximal zinc-knuckle (E-value: 6.52e^− 04^) (Fig. 1A) and the first ZnF in the ZnF-array (E-value: 1.65e^− 11^). However, as the sequence displayed nucleotide variability from the second ZnF onwards, the ZnF-array could not be resolved using the pooled sample approach.

### Variation of the PRDM9 coding minisatellite in minke whales

The variability of the minisatellite coding for the DNA-binding ZnF-array of PRDM9 was analyzed in 143 individuals, including Antarctic minke whales (*B. bonaerensis*) and two subspecies of Common minke whale - the North Atlantic (NA) minke whale (*B. acutorostrata acutorostrata)* and the North Pacific (NP) minke whale (*B. acutorostrata scammoni*). Amplification of the last exon of the *Prdm9* gene and subsequent electrophoresis on agarose gels resolved six different allele sizes (Fig. 1B), revealing that *Prdm9* shows length variation resulting from variation in the number of repeat units of the coding minisatellite. A high level of size homoplasy was observed in common minke whales and a lower level of size homoplasy in Antarctic minke whales (*Balaenoptera bonaerensis*). A length consistent with eleven 84 bp repeats was observed in all Common minke whales. In contrast, five alleles of different sizes were identified across Antarctic minke whale populations sampled in the Southern Hemisphere (Fig. 1B). The allele corresponding to a length of nine repeat units is most prevalent in Antarctic minke whales, with additional alleles with between six and ten 84 bp satellite repeat units*.*

### Diversity and diversifying selection on ZnFs of PRDM9

In addition to variation in repeat-number, sequencing revealed nucleotide diversity between minisatellite repeats. To explore this diversity, the coding minisatellite was sequenced in all individuals and all repeat units that code for individual ZnFs extracted from the translated nucleotide sequences. Based on amino acid variation within each predicted ZnF, twenty-six different ZnFs with HMMER bit scores > 17 were found (Fig. 1E and Additional File 6). Fourteen ZnFs types were found in multiple individuals and thus considered “common” ZnFs as well as twelve “unique” ZnFs, that were present only in a single individual. The most variable amino acids are 13, 16, and 19, located in positions − 1, 3 and 6 of the alpha-helix responsible for DNA binding specificity (see Fig. 1C). A mixed-effects model of evolution (MEME) analogous to [33], identified episodic diversifying selection at amino acid position 16 (Additional File 7), even when conservatively using only the fourteen common ZnFs from our dataset. Nevertheless, not all ZnFs in our dataset differ in amino acids at these three DNA-binding residues. Five ZnFs share the DNA contact residues Leucine, Asparagine and Glycine (LNG), but instead differ at amino acid positions in beta-helices flanking the cysteine residues that bind the zinc-ion (positions 3, 10 and 12). Of these, three were already found in the genomic sequences of *Balaenoptera acutorostrata scammoni* (*Ba) (Antarctic minke whale) and *Balaenoptera bonaerensis* (*Bb), the North Atlantic minke whale, as shown in Fig. 1E.

### PRDM9 ZnF-array diversity in minke whales

Both subspecies of minke whales show diversity between ZnFs, and most types of ZnF are found in both minke whale subspecies. To explore the diversity of complete ZnF arrays between individuals, all individual minisatellite-coding sequences were translated into amino-acids, and full-length ZnF domains were predicted through an HMMER algorithm [34]. Minisatellite size homoplasy equated to an identical PRDM9 ZnF array in all Common minke whale samples from the Northern Hemisphere (NH). This PRDM9 variant, NH12_A, consists of twelve ZnFs in total, the proximal zinc-knuckle and eleven ZnF in the array (Fig. 1D). In contrast, seventeen different ZnF-Domains of PRDM9 are found in samples of *Balaenoptera bonaerensis* from the Southern Hemisphere (SH) (Fig. 1D).

### Evolutionary turnover of *Prdm9* in minke whales

**Fig. 2:**
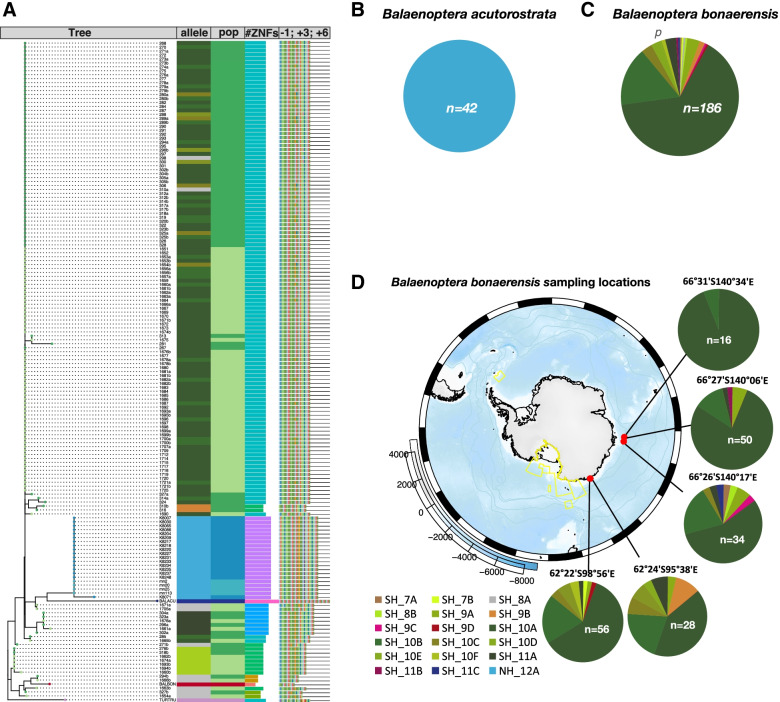
*Prdm9* Phylogenetic analyses and allele frequencies at different geographical scales. **A** PRDM9 phylogenetic analyses excluding hypervariable sites across all geographical regions, including several outgroups, from left to right Tree: Nucleotide Phylogeny of PRDM9 alleles allele: PRDM9 coding minisatellite allele colored as the translated variant from Fig. 1 pop: assigned population of individuals (dark blue): North Pacific, (light blue) North Atlantic, (light green) Antarctic Area IV, (dark green) Antarctic Area V, as well as Genome Reference alleles: (blue) *Balaenoptera acutorostrata scammoni*, (red) *Balaenoptera bonaerensis* and outgroups (pink) *Tursiops Truncatus*. # ZnFs: number of ZnFs − 1, + 3, + 6: Color coded Hypervariable positions of all repeats. **B** allelic diversity in Common minke whales (**C**) allelic diversity in Antarctic minke whales (**D**) fine-scale diversity by Sampling locations of Antarctic minke whales on a map of the Southern Ocean (yellow delineations show protected areas)

To understand the evolutionary relationships of the different minke whale species, phylogenetic reconstruction of the *Prdm9* hypervariable region of all individuals was performed. To account for the length variation between the minisatellite-like exon, the minisatellite was partitioned into its 84 bp repeat units, which also correspond to a single complete ZnFs coding unit, as shown in. Nucleotide repeats from genome references of Artiodactyla species were used as outgroups where a complete PRDM9 domain architecture with at least eight internal ZnFs was previously confirmed (Additional File 2). Distance matrices based on minimum edit-distance (Hamming) as in [35] were computed for the minisatellite-like repeats within the array (Fig. 2A), as depicted in the cartoon in Additional File 8, once including all nucleotides (Additional File 9) and again, after removing the hypervariable positions relevant for DNA binding specificity (Fig. 2A). Both *Prdm9* phylogenetic trees (Fig. 2A and Additional File 9) separate subspecies into distinct phylogenetic groups. One common minke whale variant is seen in both subspecies; North Atlantic minke whale *(B. a. acutorostrata)* and North Pacific minke whales *(B. a. scammoni)*, and the *Balaenoptera acutorostrata scammoni* reference allele clusters within the same phylogenetic branch in Fig. 2A (where hypervariable sites were removed). Similarly, the allele extracted from the genome of *Balaenoptera bonaerensis* clusters within the phylogenetic group that includes SH_8A and SH_8B alleles from our Antarctic minke whale samples. When repeat-distances of full-length alleles were used, the phylogeny of our minke whale samples is mirrored, with alleles showing slightly larger divergence time. However, when hypervariable sites are included, the reference alleles of *Baleanoptera bonarensis* and *Balaenoptera acutorostrata scammoni* no longer cluster with their subspecies and the latter is placed outside of the minke whale phylogeny, closest to the genome reference allele of the bottlenose dolphin (*Tursiops truncatus).*

### Diversity of PRDM9 at different geographical scales

The allelic diversity of *Prdm9* within each Hemisphere (Fig. 2B and C) was explored. As shown in Fig. 2B, only a single *Prdm9* allele is found in the Northern Hemisphere, inhabited by Common minke whales. In contrast, there is extensive PRDM9 diversity in the Southern Hemisphere in Antarctic minke whales (*Balaenoptera bonaerensis)*, as seen in (Fig. 2C). Variability at finer geographical scales in the Southern Hemisphere was investigated by partitioning the data into sampling locations whenever accurate catch-locations were available (Fig. 2D). The observed allelic diversity varied between Antarctic sampling sites (Fig. 2D). The most common alleles are SH_10A, and SH_10B, which occur at frequencies of 10–20% in all sampling locations, as shown in Fig. 2C. All other alleles differ between sampling locations, and at least one allele is unique to each sampling site. The highest number of alleles (ten) is seen at catching location 66°26′S140°17′E, which comprises only an average-sized sample of Antarctic minke whale individuals (*n* = 34).

### Population structure of minke whales

To understand possible evolutionary consequences of PRDM9 for minke whale speciation in the face of what may be recent secondary mixing, requires a context of levels of population isolation. Population structure was first measured across the four sample regions using *Prdm9* diversity as a marker (Table 1). Using partitioned 84 bp coding minisatellite-like repeat types of *Prdm9*, the Average Pairwise Nucleotide Diversity (APND) and basic population parameters were determined, including population θ as an unbiased estimator of population structure, [36], and Gst, the per-site distance for multiple alleles [37]. To quantify different aspects of population structure as a complementary measure, Jost’s D (D_J_), the fraction of allelic variation among populations [38], was also included, which measures mainly the differentiation of the most common alleles [36].

**Table 1:** Nucleotide diversity of the minisatellite coding for the PRDM9-ZnF array, analyzed per sample region. Average pairwise nucleotide diversity was analyzed for all sites, and also excluding the nucleotides coding for amino-acids at hypervariable sites (− 1, + 3, + 6) in the alpha-helix of ZnFs

Sample region	Area V	Area IV	North Atlantic	North Pacific
Number of analyzed 84 bp repeat units per population	554	577	170	40
Population theta (segregating sites)	2031	1731	2102	2116
Average pairwise nucleotide diversity (excluding hypervariable sites)	0.0148 ± 0.0001	0.0143 ± 0.0001	0.0184 ± 0.0002	0.0183 ± 0.0002
Average pairwise nucleotide diversity (including hypervariable sites)	0.0382 ± 0.0005	0.0374 ± 0.0005	0.0434 ± 0.0006	0.0437 ± 0.0006

The APND of repeats is similar across all sampled populations and between common minke whales and Antarctic minke whales. When the nucleotides coding for hypervariable sites (− 1, + 3, + 6) were excluded, APND decreased roughly 2.5-fold. The highest population θ using segregating sites is observed in Antarctic Area IV, compared to all other sampling locations, as seen in Table 1. Jost’s D and Gst both reveal a low degree of population differentiation, the highest differentiation being between Hemispheres. Antarctic Area V is differentiated from North Atlantic (G_ST_ = 0.0306, D_J_ = 0.3621) and North Pacific (G_ST_ = 0.0287, D_J_ = 0.3395). Similarly, differentiation between Antarctic Area IV and North Atlantic (G_ST_ = 0.0282, D_J_ = 0.3484) and North Pacific (G_ST_ = 0.0264, D_J_ = 0.3251) is seen. Within Hemispheres, little to no population differentiation is seen, neither between North Atlantic and North Pacific (G_ST_ = − 0.0038, D_J_ = − 0.0506) nor between Antarctic Areas IV and V (ANV vs ANIV, G_ST_ = − 0.0003, D_J_ = − 0.0033).

**Fig. 3:**
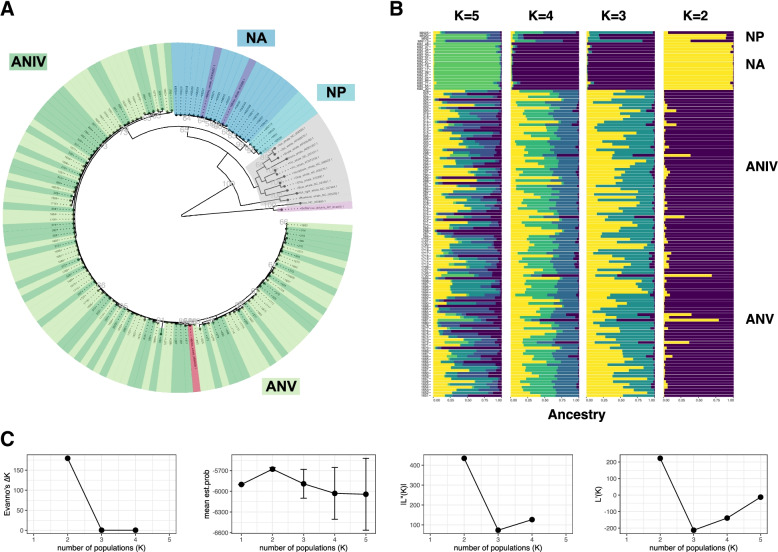
Population structure of minke whales (NP, light blue) North Pacific, (NA, dark blue) North Atlantic (ANIV, dark green) Antarctic Area IV, (ANV, light green) Antarctic Area V (**A**) Phylogeny of minke whale mitochondrial D-loop region, containing the hypervariable segments (HVS). **B** Population STRUCTURE analyses of minisatellites without a priori location information. **C** The magnitude of ∆K as a function of K, mean of the estimated probability of the data and its standard deviation (mean ± SD over 50 replicates), and Log probability of data L(K) as a function of K

A phylogeny based on the hypervariable region of the mitochondrial D-Loop (mtDNA HVR) was also constructed. The mtDNA HVR phylogenetic analyses reveal a bifurcating branch, first separating Antarctic minke whales from both Antarctic Areas from Common Minke whales (Fig. 3A). Four haplotypes are found in the North Atlantic (NA), separated with high bootstrapping support from the single mitochondrial HVR haplotype observed in North Pacific minke whales (NP) (Fig. 3A). There are multiple haplotypes found in the Southern Hemisphere, and most are shared between individuals from Antarctic areas IV and V.

To draw a more comprehensive picture of population differentiation in minke whales beyond *Prdm9* diversity, nine unlinked polymorphic autosomal microsatellite loci were also chosen for their high information content [18, 39] from [40]. Summary statistics were first applied to the microsatellite data to explore population structure using microsatellites, which revealed high heterozygosity of effectively zero FIS (Additional File 10). To avoid the scenario in which allele dropouts tend to occur, all samples had been amplified repeatedly, and genotypes with weak peaks were not accepted, thus any null alleles were most likely due to polymorphisms rather than missing data. Bayesian analysis of population structure was performed, using STRUCTURE with a recessive allele model appropriate for null alleles due to polymorphisms [41]. Figure 3B shows results using the “admixture” model without location or population information (NOLOCS) to detect only strong population structure. Implementing the Evanno method the most likely number of clusters returned by ΔK is *K* = 2 as seen in Fig. 3C, which distinguishes the two hemispheres. Increasing to *K* = *3* and *K* = *4* separates the two common minke whale subspecies from each other (as shown in Fig. 3B). Including a priori location information into the model (LOCPRIOR) can make it prone to over clustering, however, the same results are obtained (Additional File 11).

### Putative minke whale recombination initiation motifs

**Fig. 4:**
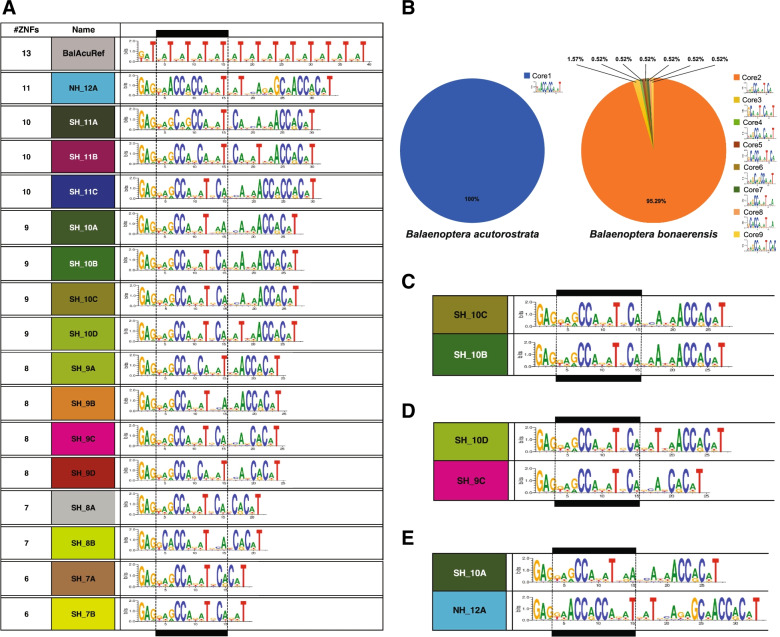
PRDM9 motif binding predictions (**A**) Array binding predictions with ZnFs#3-#6 “core motif” boxed (**B**) Pie chart of the population frequency of “core motifs” across all individuals (**C**) Identical “core motif” combinations are predicted to result in fully symmetric binding and efficient DSB formation [3, 4] (**D**) variants with somewhat dissimilar motifs, are predicted to result in some degree of asymmetry. In humans, a similar degree of motif-match still allowed DSBs necessary for successful recombination [3, 4, 29] (**E**) hypothetical combination of the most common variant of minke whale species of two hemispheres, generating a putative interspecies hybrid combination. Asymmetric positioning of recombination initiation sites would be predicted, which is implicated in F_1_-hybrid male sterility in mammals [30]

Target hotspots in humans [4, 42] and mice [28] can be predicted from C_2_H_2_ ZnF sequences using the SVM polynomial kernel model for *de-novo* binding prediction [34]. To understand the situation in minke whales, DNA binding predictions were computed. As must be true, the single PRDM9 variant found in common minke whales results in a single motif. Figure 4A shows all Common and Antarctic minke whales DNA binding motifs. Differentiation between the two minke whale species is particularly evident at ZnF#3, which is located within a “Core Motif”, based only on the ZnFs reported to be of particular importance for DNA binding [43, 44]. Despite a much larger diversity of PRDM9 motifs across Antarctic minke whales, the majority shares the same “core motif” as shown in Fig. 4B. A combination of such identical “core motif” are predicted to result in fully symmetric binding and efficient DSB formation during meiotic recombination initiation [3, 4] as shown in Fig. 4C. In contrast some degree of recombination initiation asymmetry is expected when variant combinations with somewhat dissimilar motifs come together, as depicted in Fig. 4D. In humans, a similar degree of motif-match still allowed DSBs necessary for successful recombination [3, 4, 29] Fig. 4E shows a hypothetical combination of the most common variant of both minke whale species originating from the two hemispheres, thus generating a putative interspecies hybrid combination of DNA binding motifs. These motifs do not overlap, thus predicting an asymmetric positioning of recombination initiation, which is implicated in F_1_-hybrid male sterility in mammals [30].

## Discussion

The genome reference of Minke whales and all *Artiodactyla* possess coding sequences for a complete set of KRAB, SSXRD and SET domains, a necessary feature of organisms with PRDM9-regulated recombination [32]. These proximal domains of PRDM9 are conserved across whales, just as reported in other metazoans [32], and phylogenetic reconstruction based on the proximal domains reflects the established taxonomic classification of *Artiodactyla*.

The zinc knuckle, located proximal of the ZnF array, is conserved across an extensive evolutionary timespan, as the DNA-contacting residues are identical not only in *Mysticetes* (this study) but also mice [45] rats, elephants, humans, chimpanzees, macaques and orangutans [42], with *Odontocetes* surprisingly distinct. Furthermore, the first ZnF at the start of the array is also broadly conserved, at least across *Balaenoptera.* Conservation decreases starting at the second position of the ZnF array, which alone suffices to distinguish Common minke whales from Antarctic minke whales. Evolutionary constraints thus appear to act differently on proximal and distal parts of the ZnF domain.

### PRDM9 shows high diversity, especially in Antarctic minke whales

The entire PRDM9 ZnF domain was isolated and characterized from 134 individuals from four natural populations of minke whales and discovered a total of eighteen PRDM9 variants. This high diversity is similar to that observed at the *Prdm9* gene in other vertebrate species, such as humans, mice, bovines and primates [4–6, 9, 46]. High diversity between nucleotide repeats coding for ZnFs was identified, and thus the reported lack of diversity in the reference genome of the minke whales [32] is most likely a mapping artefact. The genome was assembled from short-read and longer-read sequences of 150 bp – 20 kb [47], but the highly repetitive structure of the minisatellite coding for the zinc-finger domain likely nevertheless posed challenges for the correct assembly. This problem may be solved using novel methods for Prdm9 minisatellite assembly from long-read sequencing data [48].

Phylogenetic analysis of minisatellite repeat structures is challenging due to their rapid rate of evolution, and commonly used stepwise mutation models are based on microsatellites and typically give only a poor fit to minisatellite evolution [49]. Minisatellites, including the coding *Prdm9* minisatellite, mainly evolve by unequal crossing-over and gene conversion in meiosis [50]. A novel approach to *Prdm9* phylogenetic reconstruction was applied for minke whales, which is based on computing Hamming distances between minisatellite repeat units pioneered by [35], before constructing a phylogeny. This *Prdm9* phylogenetic reconstruction suggests that the common minke whale *Prdm9* allele appeared more recently and evolved mainly by an increase in repeat-copy after splitting from the Antarctic minke whale, which fits well with the reported evolutionary history of these minke whale species [51].

The samples used in this study were collected 40 years ago (1980–84), which represents about two to three generations of minke whales, given a typical generation time of 15–20 years [47]. Even giving the rapid evolutionary turnover of PRDM9, three generations should not have significantly increased diversity in minke whales. However, PRDM9 diversity may have decreased, as a decline in minke whales have been reported since the time our samples were collected [52, 53], and thus particularly rare PRDM9 variants, unique to specific sampling locations, may have been lost from Antarctic Minke whales.

### Population genetic analyses of minke whales and potential speciation

Taxonomists have separated the common minke whale into two subspecies, the North Atlantic minke whale (*B. a. acutorostrata*) and the North Pacific minke whale (*B. a. scammoni),* which diverged approximately 1.5 million years ago [51]. In our study, the variability between microsatellite markers reveals weak population structure and mtDNA differentiation between North Atlantic and North Pacific minke whale samples. Even though diversity between repeat types can be observed, all individuals of both Common minke whale subspecies had the identical *Prdm9* allele. Chimpanzees and bonobos are similarly closely related and share some admixture – yet despite a report of amino-acid conservation of a putative ancestral PRDM9 variant [54], bonobos and chimpanzees do not generally share *Prdm9* alleles, and both show extensive *Prdm9* diversity [46].

Identical ZnF domains suggest that subspecies should still be able to interbreed if given a chance. It is unclear whether occasional hybridization events have always occurred or are a recent phenomenon driven by anthropogenic changes, including climate change [18]. Aberrant migration patterns and northward changes in distribution of baleen whales inhabiting the North Atlantic Ocean have been observed particularly in the last decade [55]. While the permanent polar ice still upholds the geographical isolation of the two subspecies of common minke whales, allopatric speciation may be promoted. However, due to global warming, the two subspecies might come into secondary contact again in the future. In the last decades, following the accelerated sea ice loss, the Atlantic and Pacific Ocean Basins are connected for extended periods each year, making an increased inter-basin movement of minke whales more likely [56]. The removal of this geographical barrier could therefore disrupt speciation and signify the start of the breakdown of genetic isolation, especially in light of identical ZnF domains encoded by the mammalian hybrid sterility gene *Prdm9*.

### *Prdm9* diversity is not equally abundant in both hemispheres

Antarctic minke whales show much greater diversity between repeat types and complete ZnF domains, even at a fine geographical scale. Here, little population structure is evident from microsatellite data, and whales sampled in Antarctic Areas share mitochondrial haplotypes. The low levels of population differentiation between *Prdm9* repeat units in the Antarctic areas contrast with the exceptionally high levels of genetic diversity of *Prdm9* alleles in this Hemisphere. This contrasting pattern between protein-level conservation and nucleotide differentiation is fascinating and may point to functional constraints operating on different levels of PRDM9 evolution.

### Hybrids between Antarctic minke whale and common minke whale

Analysis of the hypervariable region (HVR) on the mitochondrial D-Loop suggests that the Antarctic minke whale and Common minke whale evolved from a common ancestor in the Southern Hemisphere during a period of global warming approximately 5 million years ago [51]. Common minke whales and Antarctic minke whales are now separated by both geography and seasonality. However, while their winter habitats and breeding grounds remain unknown [52], it is assumed that *B. a. acutorostrata* migrates south between November and March to give birth in warmer waters and are seen occasionally as far south as the Gulf of Mexico [57].

Interbreeding between the Northern and Southern Hemisphere appears unlikely at the time that our samples were collected (the 1980s) because our study confirms previous findings that common minke whales and Antarctic minke whales exhibit genetic differentiation in their mtDNA haplotypes [58]. Microsatellite data shows no evidence for inbreeding, and given that these species occur in geographically isolated groups that are separated by Hemispheres, and have asynchronous breeding seasons, a low probability for incomplete lineage sorting should be assumed. Our population structure analyses based on microsatellites supports a clear separation of these two species with distinct clusters separating the Hemispheres, even without including location information into the model, which can make it prone to over clustering. Our phylogenetic analysis of the last exon of the *Prdm9* gene also fits well within this evolutionary history.

Even though many hybrid incompatibilities exist [19], the *Prdm9* gene remains the only hybrid sterility gene known in vertebrates to date [21]. Fast evolutionary turnover of the coding minisatellite is seen in all mammals characterized to date [3–11, 35, 59–66] including minke whales (*this study*). One consequence of genetic variation in the coding minisatellite is that when the DNA-binding ZnF-domain changes, variation in entire species’ recombination landscapes is introduced with every change in the DNA-binding ZnF domain. Different ZnF domains, which differ even within populations of the same species [4, 23], can target different recombination hotspots [3, 4, 28, 60]. Based on *in-silico* predicted PRDM9 ZnF binding motifs found in Common minke whales and Antarctic minke whales, motifs are differentiated between species inhabiting different Hemispheres. We, therefore, speculate that the recombination initiation landscapes of the two variants of minke whales would not overlap.

Furthermore, the absence of PRDM9 diversity in the Northern Hemisphere would predict extensive historical erosion of PRDM9 binding sites in the genomes of Common Minke Whales since the split from Antarctic minke whales. Some degree of erosion may also occur when most animals share identical “core motifs”. As “core motifs” that are based on the ZnFs that appear particularly important for DNA binding are also shared by the majority of Antarctic minke whales in our dataset, a degree of PRDM9 binding site erosion would also be predicted in genomes of minke whales inhabiting the Southern Hemisphere. Together erosion on both genomes should result in non-overlapping minke whale PRDM9 binding motifs over time, a prerequisite of asymmetric PRDM9 recombination initiation.

Hybridization events between Common minke whales and Antarctic minke whales have been reported [16, 17], which shows that interbreeding and even backcrossing [17] is possible. However, PRDM9 mediated hybrid sterility in mice follows Haldane’s rule [67–69], which states that the heterogametic sex will be affected first. Yet, both reported hybrids were female [16, 17], which is the homogametic sex in minke whales. There are reports of a *Bos indicus* PRDM9 variant that, when introgressed into Holstein cattle, induced incompatibility of recombination hotspots and infertility in males but at the same time improved fertility of female hybrids [66]. These observations in cattle, and the lack of male minke whale hybrids, do not allow any conclusions as to whether the postzygotic isolation mechanisms related to PRDM9 incompatibility do or do not generally operate in minke whales. Therefore, the question remains whether PRDM9 mediated reproductive isolation mechanism exists in minke whales and whether sporadically occurring hybridization events will generate infertile males. Further studies, particularly on minke whale hybrids, are necessary to elucidate this matter.

## Conclusion

The evolutionary context of PRDM9 across even-toed ungulates was established, and the variability of the DNA-binding domain of PRDM9 was characterized in detail across different populations of minke whales from the Southern Oceans and the North Atlantic and North Pacific, overall - and at different geographical scales. Sequencing revealed rapid evolutionary turnover of the minisatellite encoding the ZnF array of PRDM9 and evidence of episodic diversifying selection on an amino-acids that is important for DNA-binding specificity. In the Southern Hemisphere, the extensive PRDM9 protein diversity poses an apparent contradiction to the low levels of population structure observed in the same individuals. In contrast, maintenance of conserved protein sequence even across minke whale subspecies boundaries is observed in common minke whales inhabiting the Northern Hemisphere.

## Methods

### PRDM9 occurrence and protein domain prediction in diverse taxa

To infer PRDM9 occurrence in Artiodactyla, the annotated protein XP_028019884.1 from *Balaenoptera acutorostrata scammoni* as the query protein with exonerate [70] (v2.2.0) and the --protein2genome model to extract the best hit, in all publicly available genomic resources of Artiodactyla (Additional File 1). InterProScan [71] (v5.46–81.0) and HMMER3 [72] (v3.3) were then used to create a curated dataset of PRDM9 orthologues, which contained the KRAB, SSXRD, and SET domains. Subsequently, for each species, the extracted coding sequences (CDS) were translated and investigated using KRAB, SSXRD, SET and ZnF as bait to obtain the protein domain architecture.

### Phylogenetic analyses of PRDM9 protein domain architecture

For the phylogenetic reconstruction across *Artiodactyls* (Additional File 3), the amino-acid sequence of only the KRAB, SSXRD, and SET domains were used. These protein domains were concatenated and used as input for the software BAli-phy [73] (v3.5.0). BAli-phy was run twice with 10,000 iterations each and the default settings for amino acid input. Subsequently, the majority consensus tree was obtained by skipping the first 10% of trees as burn-in, rooted on the branch outside the Whippomorpha and visualized in Figtree (http://tree.bio.ed.ac.uk/software/figtree/; v1.4.4).

### Long-range sequencing of the *Prdm9* gene

The full-length DNA sequence of the PRDM9 protein was extracted from the UCSC genome browser minke whale assembly, and primers to flank the entire protein-coding portion, encompassing the KRAB, SSXDR, PR/SET and ZnF binding domains, were designed using Primer-BLAST [74] (Additional File 12). The *Balaenoptera acutorostrata Prdm9* gene, including all introns and exons, was amplified across a ~ 11 kb interval by long-range PCR. The entire interval was then sequenced using phased long-range Nanopore Sequencing with MinION (Oxford Nanopore). Whole-length consensus sequences generated from 639 sequencing reads cover the entire 10,582 bp, with an average per base pair coverage of 269x. Due to the high sequence error rate of Nanopore sequencing – mostly nucleotide dropout – the single-read accuracy is very low. However, having achieved high per base pair coverage (>200x) any random errors should be cancelled out sufficiently to generate an accurate consensus sequence.

This consensus sequence was used as reference for *in-silico* predictions of functional domains and mapped human PRDM9 Exons 3–11 from ENSEMBL (ENST00000296682.3). By manually splicing all unaligned sequence fragments, an *in-silico* predicted mRNA of *Balaenoptera acutorostrata Prdm9* was generated. This sequence was then submitted as ‘.fasta’ to the Entrez Conserved Domains Database (CDD) home page, (https://www.ncbi.nlm.nih.gov/Structure/cdd/wrpsb.cgi) with the following parameters; searching against Database CDD v3.18–52,910 PSSMs, expect value threshold: 0,01, without low-complexity filter, composition-based statistics adjustment, rescuing borderline hits (ON) a maximum number of 500 hits and concise result mode).

### Wild minke whale samples

Performed investigations are not considered to be animal trials under the German animal welfare act, since samples were obtained from commercial whaling between 1980 and 1984. A total of *n* = 143 DNA samples of minke whale with incomplete information about the subspecies from four different defined commercial whaling areas. These include North Atlantic (NA; *n* = 17) and North Pacific (NP; *n* = 4) individuals captured during migration season, as well as Antarctic Ocean Areas IV (AN IV; *n* = 65) and V (AN V; *n* = 57) were obtained during feeding season. A subset of these samples had been used in van Pijlen et al., 1995 [58]. Samples from the Antarctic areas included five duplicate samples (318, 325, 327, 1661, 1663), which were used as internal controls, analyzed them for all parameters, and then excluded duplicate measurements from the dataset after identical results had been confirmed. (Both the full dataset and the parsed dataset are available as Additional Files). For the detailed DNA-extraction protocol, see [58]. Briefly, genomic DNA was extracted from the skin (NA) and muscle biopsies (NP, AN) with Phenol-Chlorophorm and stored in TE at − 80 °C in the 1980s. If the DNA was dried out, it was first eluted with TE. All DNA concentrations were determined with fluorescent Nanodrop-1000 (Thermo Fisher Scientific). For all performed analysis, 20 ng/μl DNA working stocks were prepared and stored at − 20 °C.

### *Prdm9* coding minisatellite array PCR and sequencing

To characterize the minisatellite-coding for the ZnF array in more detail, primers were designed to nest between the first two conserved ZnFs were designed. The reverse primer was identical to the long-range amplification primer distal to the coding sequence for the ZnF array. The minisatellite coding for the ZnF array of PRDM9 of 143 Balaenoptera individuals was amplified from 20 ng genomic DNA in a 20 μl PCR reaction optimized for the amplification of minisatellites. With 0.5 mM primers that were designed for this study using the *Balaenoptera acutorostrata scammoni* (XM_007172595) reference and included the PRDM9 minisatellite-like ZnF array and additional 100 bp flanking regions at 5′ and 3′. Primers Prdm9ZnFA_Bal_R: and Prdm9ZnFA_Bal_F (Additional File 12) and 1x AJ-PCR Buffer described in [75] and 0.025 U/μl Taq-Polymerase and 0.0033 U/μl Pfu-Polymerase. Cycling conditions were: initial denaturation at 95 °C for 1:30 min followed by 33 cycles including 96 °C 15 s, 61 °C 20 s and 70 °C 2:00 min and finally 70 °C 5 min, and hold at 4 °C in a Veriti Thermal Cycler (Applied Biosystems). Agarose gel electrophoresis 1.5% Top Vision Low Melting Point Agarose gel (Thermo Fisher Scientific) with SYBR Safe (Thermo Fisher Scientific) was used to visualize allele sizes as well as zygosity. All bands were excised from the gel (Molecular Imager® Gel Doc™ XR System with Xcita-Blue™ Conversion Screen (Biorad)), and recovered with 2 U/100 mg Agarase. If the individuals were homozygous, the extracted DNA was directly Sanger sequenced from in 5′ and 3′ directions for each sample with the BigDye™ Terminator v3.1 Cycle Sequencing Kit (Thermo Fisher Scientific) according to the manufacturer’s protocol with the same primers as for the amplification (Prdm9ZnFa_Bal_R/Prdm9ZnFa_Bal_F). The sequencing-reaction was carried out in the ABI 16-capillary 3130xl Analyzer (Applied Biosystems). Heterozygous samples were subcloned before sequencing.

### Subcloning of heterozygous alleles of identical lengths

Subcloning was performed for a subset of samples when Sanger sequencing revealed heterozygous alleles of the same length that could not be distinguished by electrophoresis, but revealed heterozygous nucleotides in the chromatogram. Thus, the remaining PCR product was cloned into TOPO TA (Invitrogen) vectors and transferred the vectors into OneShotTop10 chemically competent cells (Invitrogen). All steps were carried out according to the manufacturer’s manuals. Eight positive clones were picked for each sample, and the DNA was extracted in HPLC-grade water at 96 °C for 10 Minutes. The cell debris, was removed by centrifugation and the supernatant was directly used for PCR, gel-purification and Sanger-sequencing as described in the section above.

### *Prdm9* coding minisatellite repeat diversity

Different alleles and numbers of repeat units per array were determined, by DNA sequencing. Sanger-reads were *de-novo* assembled by Geneious Software 10.2.3 [76]. Taking each repeat unit as an individual allele, all non-unique alleles were stacked and the mutation rate per base pair per generation (population θ), and average pairwise nucleotide diversity were computed with the R package “pegas” [77]. The latter was calculated in two ways: (i) using the entire minisatellite-like repeat sequence; (ii) after removing nucleotides coding for the hypervariable sites that translate into the DNA binding positions of individual ZnF.

### Phylogenetic analyses of the *Prdm9* hypervariable region

The R package RepeatR, was developed specifically for this publication to generate distance matrices based on pairwise Hamming (i.e. minimum edit) distances between all *Prdm9* minisatellites repeat units by applying specific weighting costs as given in Vara et al. 2019 [35]. In brief, for each possible repeat combination (r, r’) the hamming distances of the corresponding repeat units r = (r1; r2; r3; …) and r’ = (r’ 1; r’ 2; r’ 3; …) were used to derive the edit distance between r and r’. Before calculating the edit distance, the codons coding for the hypervariable amino acid positions (− 1, + 3, + 6) were removed for each repeat unit, and the weighting cost of w_mut_ = 1, w_indel_ = 3.5 and w_slippage_ = 1.75 as given in [35].

A neighbor-joining tree was calculated with the bionj function of the R package ape [78], and rooted on the branch leading to the bottlenose dolphin (*Tursiops truncatus*) and visualized in Figtree (http://tree.bio.ed.ac.uk/software/figtree/ v1.4.4).

### PRDM9 ZnF array coding sequence dN/dS analysis

ZnFs were obtained by translating the consensus sequences into the corresponding protein variants. Only the internal non-unique ZnFs were then extracted and stacked, before determining episodic diversifying selection among Zinc-fingers determined by a mixed-effects model of evolution (MEME) at (https://www.datamonkey.org) as described in [33].

### Prediction of DNA-binding motifs of different PRDM9 variants

DNA-binding Specificities of the different Cys_2_His_2_ Zinc Finger domain variants were predicted in-silico using the SVM polynomial kernel method within “Princeton ZnF” (http://zf.princeton.edu/) [34].

### STR genotyping

Nine autosomal, as well as X/Y microsatellite loci with di- or tetramer repeat motifs, were analyzed for all samples: EV001, EV037 [39], GATA028, GATA098, GATA417 [79], GT023, GT310, GT509, GT575 [80] and sex loci X and Y [81]. Four separate multiplexing reactions were performed for each individual, and each contained 40 ng of DNA, 0.2 μM of each primer, 5 mM Multiplex-Kit (Qiagen) and HPLC water to a total volume of 10 μL per sample. Primers (Additional File 12) were purchased from Sigma Aldrich; the reverse Primers were tagged at their 5′ end with fluorescent tags (HEX, FAM or JOE). The amplification conditions were denaturation at 95 °C for 15:30 min, annealing 1:30 min and elongation at 72 °C for 11:30 min. The annealing temperatures were: 59 °C for Multiplex 1 with GT023 (HEX), EV037 (HEX) (FAM), and 54 °C for both Multiplex 2, with GT575 (HEX), GATA028 (FAM), and Multiplex 3 with GATA098 (FAM), GT509 (FAM) and GATA417 (JOE). The annealing temperature of 60 °C was used for Multiplex 4, which included GT310 (HEX) and EV001 (JOE). The reactions were diluted with 100 μl water (HPLC grade) after amplification. One microliter of the diluted product was added to 10 μl of 100:1 mixture of HiDi Formamide (Thermo Fisher Scientific) and Genescan ROX_500_ dye size standard (Thermo Fisher Scientific). Fragment analyses were carried out on the 16-capillary electrophoresis system ABI 3300 Genetic Analyzer (Applied Biosystems).

### STR analysis

MSA analysis [65] was performed using standard parameters, which calculated Weinberg expectation (Fis), Shannon Index (Hs), allele numbers (A) and allele sizes. To detect both weak and strong population structures, simulations with and without LOCPRIOR and USEPOPINFO were run, respectively. For all simulations, the more conservative “correlated allele frequencies” -model was used, which assumes a level of non-independence. To ensure that a sufficient number of steps and runs have been performed, using a burn-in period of 1.000 and runs of 100.000 Markov Chain Monte-Carlo (MCMC) repeats for both types of simulations, each for 50 iterations for successive K values from 1 to 10 [82].. The a-priori location to be able to detect even weak population differentiation was also used. In both datasets, the web-based STRUCTURE Harvester software was used [83] to determine the rate of change in the log probability between successive K values via the ad-hoc statistic ΔK from [84]. Figures were rendered using STRUCTURE PLOT V2.0 [85].

### Sequencing of the mitochondrial hypervariable region on the D-loop

The noncoding mtDNA-D-Loop region of 143 individuals was amplified in two overlapping PCR reactions, as described in [40]. PCR amplification of two different lengths fragments was performed for each individual: 1066 bp and 331 bp followed by sequencing the longer product in forward and the shorter in the reverse direction. The used primers (Additional File 12) were MT4 (M13F) and MT3 (M13R) for the longer product, and BP15851 (M13F) and MN312 (M13R) for the shorter PCR product from [40]. The 10 μl reaction was carried out with 40 ng genomic DNA and 0.2 μM of each primer and 5 mM Multiplex PCR Kit (Qiagen). Cycling conditions were identical for both directions with 95 °C 15:30 min, 53 °C 1:30 min, 72 °C 13:30 min and hold at 4 °C in a Veriti Thermal Cycler (Applied Biosystems). The PCR products were purified with 3 μl Exo/SAP and then cycle-sequenced with the BigDye™ Terminator v3.1 Cycle Sequencing Kit (Thermo Fisher Scientific) according to manufacturer instructions with BP15851 (M13F) for the forward PCR product and MN312 (M13R) for the reverse PCR product, respectively. The mixes were then purified with BigDye X-Terminator™ Purification Kit (Thermo Fisher Scientific) and sequenced by capillary electrophoresis on an ABI 16-capillary 3130xl Analyzer (Applied Biosystems). The sequences were *de-novo* assembled, and consensus sequences were generated with Geneious Software 10.2.3 [76].

### Phylogenetic analyses of the mitochondrial D-loop

The phylogenetic tree from the mitochondrial D-loop region was reconstructed of all species from the infraorder *Cetacea* (Brisson, 1762), where public genomic resources were available with reference sequence locations given in Additional File 13. The corresponding D-loop was aligned with MAFFT version 5 [86](v7.471) with the L-INS-i algorithm and manually curated. The maximum-likelihood tree was calculated under the TN + F + I + G4 model using IQ-TREE [87] (v1.6.12) mid-point rooted and visualized in Figtree (http://tree.bio.ed.ac.uk/software/figtree/; v1.4.4).

## Supplementary Information

**Additional File 1.** Public genome resources for PRDM9.

**Additional File 2.** PRDM9 occurrence and protein domain prediction with InterProScan.

**Additional File 3. **PRDM9 Phylogenetic analysis across *Artiodactyla.*

**Additional File 4. **Alignment of PRDM9 zinc knuckles from *Artiodactyla*.

**Additional File 5. **Alignment of the first ZnF in the ZNF array of *Artiodactyla.*

**Additional File 6.** All PRDM9 ZnF types in minke whales.

**Additional File 7.** Signals of selection on amino-acid ZnF identified in this study.

**Additional File 8.** Methodological approach.

**Additional File 9. ***Prdm9* Phylogenetic analyses including hypervariable sites.

**Additional File 10.** Descriptive statistics of microsatellites.

**Additional File 11.** Population structure analyses on a set of ten hypervariable microsatellite loci.

**Additional File 12.** Primers used in this study.

**Additional File 13.** Public mitochondrial resources.

**Additional File 14.** Uncropped blot of the image used in Fig. 1.

## Data Availability

Most data generated or analyzed during this study are included in this published article and as Additional files, genetic data generated and analyzed during the current study are available in the Zenodo repository, under 10.5281/zenodo.4309436 and R Scripts are available from https://gitlab.gwdg.de/mpievolbio-it/repeatr.

## Abbreviations

AN IV: Antarctic Area IV
AN V: Antarctic Area V
APND: Average Pairwise Nucleotide Diversity
CDS: coding sequences
HMMER: Hidden Markov Model by Eddi Rivas
HVR: hypervariable region on the mitochondrial D-loop
KRAB: Kruppel-associated box- domain
MCMC: Markov Chain Monte-Carlo
MEME: Mixed-effects model of evolution
mtDNA: mitochondrial deoxyribonucleic acid
NA: North Atlantic
NH: Northern Hemisphere
NP: North Pacific
PRDM9: PR-domain containing 9 protein
SET: Su(var)3–9, Enhancer-of-zeste and Trithorax
SH: Southern Hemisphere
SSXRD: SSX-repression-domain
STR: Short tandem repeat
SVM: Support vector machines
ZnF: zinc-fingers of Cystin_2_Histidin_2_ type
