# Supplementary material for: Evolution of the recombination regulator PRDM9 in minke whales
Source: BMC Genomics. 2022 Mar 16;23:212. doi: 10.1186/s12864-022-08305-1 (PMC8925151; doi:10.1186/s12864-022-08305-1)
Supplement: Supplementary file 1 — Additional File 1. Public genome resources for PRDM9. [file 12864_2022_8305_MOESM1_ESM.docx]

| URL-GENOME | https://ftp.ncbi.nlm.nih.gov/genomes/all/GCF/000/493/695/GCF_000493695.1_BalAcu1.0/GCF_000493695.1_BalAcu1.0_genomic.fna.gz | https://ftp.ncbi.nlm.nih.gov/genomes/all/GCA/000/978/805/GCA_000978805.1_ASM97880v1/GCA_000978805.1_ASM97880v1_genomic.fna.gz | https://ftp.ncbi.nlm.nih.gov/genomes/all/GCA/009/873/245/GCA_009873245.2_mBalMus1.v2/GCA_009873245.2_mBalMus1.v2_genomic.fna.gz | http://alfred.liv.ac.uk/downloads/bowhead_whale/bowhead_whale_scaffolds.zip | https://ftp.ncbi.nlm.nih.gov/genomes/all/GCF/002/263/795/GCF_002263795.1_ARS-UCD1.2/GCF_002263795.1_ARS-UCD1.2_genomic.fna.gz | https://ftp.ncbi.nlm.nih.gov/genomes/all/GCF/000/803/125/GCF_000803125.2_CamDro3/GCF_000803125.2_CamDro3_genomic.fna.gz | https://ftp.ncbi.nlm.nih.gov/genomes/all/GCA/004/024/745/GCA_004024745.2_CatWag_v2_BIUU_UCD/GCA_004024745.2_CatWag_v2_BIUU_UCD_genomic.fna.gz | ftp://ftp.ensembl.org/pub/release-100/fasta/delphinapterus_leucas/dna/Delphinapterus_leucas.ASM228892v3.dna.toplevel.fa.gz | https://ftp.ncbi.nlm.nih.gov/genomes/all/GCA/002/189/225/GCA_002189225.1_ASM218922v1/GCA_002189225.1_ASM218922v1_genomic.fna.gz | https://ftp.ncbi.nlm.nih.gov/genomes/all/GCA/004/363/455/GCA_004363455.1_EubJap_v1_BIUU/GCA_004363455.1_EubJap_v1_BIUU_genomic.fna.gz | https://ftp.ncbi.nlm.nih.gov/genomes/all/GCF/006/547/405/GCF_006547405.1_ASM654740v1/GCF_006547405.1_ASM654740v1_genomic.fna.gz | https://ftp.ncbi.nlm.nih.gov/genomes/all/GCA/004/027/065/GCA_004027065.2_HipAmp_v2_BIUU_UCD/GCA_004027065.2_HipAmp_v2_BIUU_UCD_genomic.fna.gz | https://ftp.ncbi.nlm.nih.gov/genomes/all/GCA/004/363/515/GCA_004363515.1_IniGeo_v1_BIUU/GCA_004363515.1_IniGeo_v1_BIUU_genomic.fna.gz | https://ftp.ncbi.nlm.nih.gov/genomes/all/GCF/003/676/395/GCF_003676395.1_ASM367639v1/GCF_003676395.1_ASM367639v1_genomic.fna.gz | https://ftp.ncbi.nlm.nih.gov/genomes/all/GCF/000/442/215/GCF_000442215.1_Lipotes_vexillifer_v1/GCF_000442215.1_Lipotes_vexillifer_v1_genomic.fna.gz | https://ftp.ncbi.nlm.nih.gov/genomes/all/GCA/004/329/385/GCA_004329385.1_megNov1/GCA_004329385.1_megNov1_genomic.fna.gz | https://ftp.ncbi.nlm.nih.gov/genomes/all/GCA/004/027/085/GCA_004027085.1_MesBid_v1_BIUU/GCA_004027085.1_MesBid_v1_BIUU_genomic.fna.gz | https://ftp.ncbi.nlm.nih.gov/genomes/all/GCF/005/190/385/GCF_005190385.1_NGI_Narwhal_1/GCF_005190385.1_NGI_Narwhal_1_genomic.fna.gz | https://ftp.ncbi.nlm.nih.gov/genomes/all/GCF/003/031/525/GCF_003031525.1_Neophocaena_asiaeorientalis_V1/GCF_003031525.1_Neophocaena_asiaeorientalis_V1_genomic.fna.gz | https://ftp.ncbi.nlm.nih.gov/genomes/all/GCF/000/331/955/GCF_000331955.2_Oorc_1.1/GCF_000331955.2_Oorc_1.1_genomic.fna.gz | https://ftp.ncbi.nlm.nih.gov/genomes/all/GCF/002/742/125/GCF_002742125.1_Oar_Rambouillet_v1.0/GCF_002742125.1_Oar_Rambouillet_v1.0_genomic.fna.gz | https://ftp.ncbi.nlm.nih.gov/genomes/all/GCA/003/071/005/GCA_003071005.1_ASM307100v1/GCA_003071005.1_ASM307100v1_genomic.fna.gz | https://ftp.ncbi.nlm.nih.gov/genomes/all/GCF/002/837/175/GCF_002837175.2_ASM283717v2/GCF_002837175.2_ASM283717v2_genomic.fna.gz | https://ftp.ncbi.nlm.nih.gov/genomes/all/GCF/000/003/025/GCF_000003025.6_Sscrofa11.1/GCF_000003025.6_Sscrofa11.1_genomic.fna.gz | https://ftp.ncbi.nlm.nih.gov/genomes/all/GCA/006/408/655/GCA_006408655.1_LMD/GCA_006408655.1_LMD_genomic.fna.gz | https://ftp.ncbi.nlm.nih.gov/genomes/all/GCA/003/227/395/GCA_003227395.1_ASM322739v1/GCA_003227395.1_ASM322739v1_genomic.fna.gz | https://ftp.ncbi.nlm.nih.gov/genomes/all/GCF/011/762/595/GCF_011762595.1_mTurTru1.mat.Y/GCF_011762595.1_mTurTru1.mat.Y_genomic.fna.gz | https://ftp.ncbi.nlm.nih.gov/genomes/all/GCF/000/164/845/GCF_000164845.3_VicPac3.1/GCF_000164845.3_VicPac3.1_genomic.fna.gz | https://ftp.ncbi.nlm.nih.gov/genomes/all/GCA/004/364/475/GCA_004364475.1_ZipCav_v1_BIUU/GCA_004364475.1_ZipCav_v1_BIUU_genomic.fna.gz |
| --- | --- | --- | --- | --- | --- | --- | --- | --- | --- | --- | --- | --- | --- | --- | --- | --- | --- | --- | --- | --- | --- | --- | --- | --- | --- | --- | --- | --- | --- |
| SPECIES | Balaenoptera acutorostrata acutorostrata | Balaenoptera bonarensis | Balaenoptera musculus | Balaena mysticetus | Bos taurus | Camelus dromedarius | Catagonus wagneri | Delphinapterus leucas | Eschrichtius robustus | Eubalaena japonica | Globicephala melas | Hippopotamus amphibius | Inia geoffrensis | Lagenorhynchus obliquidens | Lipotes vexillifer | Megaptera novaeangliae | Mesoplodon bidens | Monodon monoceros | Neophocaena asiaeorientalis asiaeorientalis | Orcinus orca | Ovis aries | Phocoena phocoena | Physeter catodon | Sus scrofa | Tragulus kanchil | Tursiops aduncus | Tursiops truncatus | Vicugna pacos | Ziphius cavirostris |
