# Supplementary material for: Evolution of the recombination regulator PRDM9 in minke whales
Source: BMC Genomics. 2022 Mar 16;23:212. doi: 10.1186/s12864-022-08305-1 (PMC8925151; doi:10.1186/s12864-022-08305-1)
Supplement: Supplementary file 2 — Additional File 2. PRDM9 occurrence and protein domain prediction with InterProScan. [file 12864_2022_8305_MOESM2_ESM.docx]

| Species | Common name | Alias | Taxid | | KRAB | SSXRD | SET | ZnF #1 | ZnF array |
| --- | --- | --- | --- | --- | --- | --- | --- | --- | --- |
| Balaenoptera acutorostrata | Minke whale | Balacu | | 9767 | X | X | X | X | 13(1) |
| Balaenoptera bonarensis | Antarctic minke whale | Balbon | | 33556 | X | X | X | X | 4(2) |
| Balaenoptera musculus | Blue whale | Balmus | | 9771 | - | X | X | X | 5(2) |
| Balaena mysticetus | Bowhead whale | Balmys | | 27602 | X | X | X | X | 3(2) |
| Bos taurus | Cattle | Bostau | | 9913 | X | X | X | X | 13(1) |
| Camelus dromedarius | Arabian camel | Camdro | | 9838 | X | X | X | X | 4(1) |
| Catagonus wagneri | Chacoan peccary | Catwag | | 51154 | X | X | X | X | 7 |
| Delphinapterus leucas | Beluga whale | Delleu | | 9749 | X | X | X | X | 2(1) |
| Eschrichtius robustus | Grey whale | Escrob | | 9764 | X | X | X | X | 2(3) |
| Eubalaena japonica | North pacific right whale | Eubjap | | 302098 | X | X | X | X | 2(2) |
| Globicephala melas | Long-finned pilot whale | Glomel | | 9731 | X | X | X | X | 3(1) |
| Hippopotamus amphibius | Hippopotamus | Hipamp | | 9833 | X | X | X | - | - |
| Inia geoffrensis | Boutu | Inigeo | | 9725 | X | X | X | X | 3(2) |
| Lagenorhynchus obliquidens | Pacific white-sided dolphin | Lagobl | | 90247 | X | X | X | X | 1(2) |
| Lipotes vexillifer | Yangtze river dolphin | Lipvex | | 118797 | X | X | X | X | 3(2) |
| Megaptera novaeangliae | Humpback whale | Megnov | | 9773 | X | X | X | X | 4(2) |
| Mesoplodon bidens | Sowerby's beaked whale | Mesbid | | 48745 | X | X | X | - | 4(1) |
| Monodon monoceros | Narwhal | Monmon | | 40151 | X | X | X | X | 1(1) |
| Neophocaena asiaeorientalis asiaeorientalis | Yangtze finless porpoise | Neoasi | | 1706337 | X | X | X | X | 6 |
| Orcinus orca | Killer whale | Orcorc | | 9733 | X | X | X | X | 3(1) |
| Ovis aries | Sheep | Oviari | | 9940 | X | X | X | X | 13(1) |
| Phocoena phocoena | Harbor porpoise | Phopho | | 9742 | X | X | X | X | 2 |
| Physeter catodon | Sperm whale | Phycat | | 9755 | X | X | X | X | 4(1) |
| Sus scrofa | Pig | Susscr | | 9823 | X | X | X | X | 9 |
| Tragulus kanchil | Lesser mouse-deer | Trakan | | 1088131 | X | X | X | X | 5(1) |
| Tursiops aduncus | Indopacific bottlenose dolphin | Turadu | | 79784 | X | X | X | X | 1(1) |
| Tursiops truncatus | Common bottlenose dolphin | Turtru | | 9739 | X | X | X | X | 8(1) |
| Vicugna pacos | Alpaca | Vicpac | | 30538 | X | X | X | X | 4(1) |
| Ziphius cavirostris | Cuvier's beaked whale | Zipcav | | 9760 | X | X | X | - | 4(1) |
