# Supplementary figures and images for: Evolution of the recombination regulator PRDM9 in minke whales
Source: BMC Genomics. 2022 Mar 16;23:212. doi: 10.1186/s12864-022-08305-1 (PMC8925151; doi:10.1186/s12864-022-08305-1)

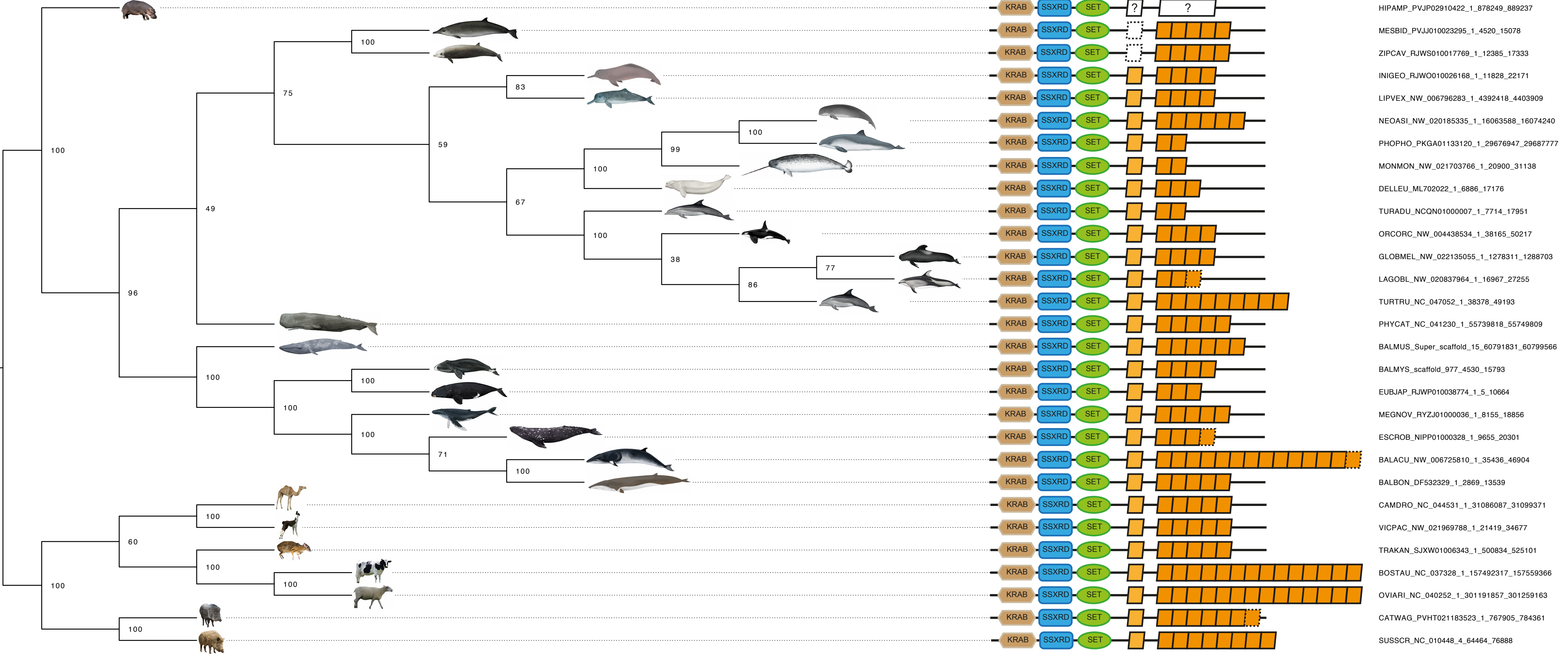

Supplement: Supplementary file 3 — Additional File 3. PRDM9 Phylogenetic analysis across Artiodactyla. [file 12864_2022_8305_MOESM3_ESM.pdf]

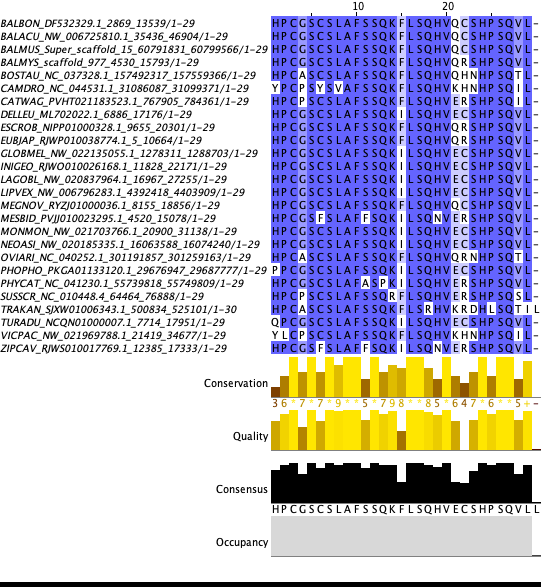

Supplement: Supplementary file 4 — Additional File 4. Alignment of PRDM9 zinc knuckles from Artiodactyla. [file 12864_2022_8305_MOESM4_ESM.png]

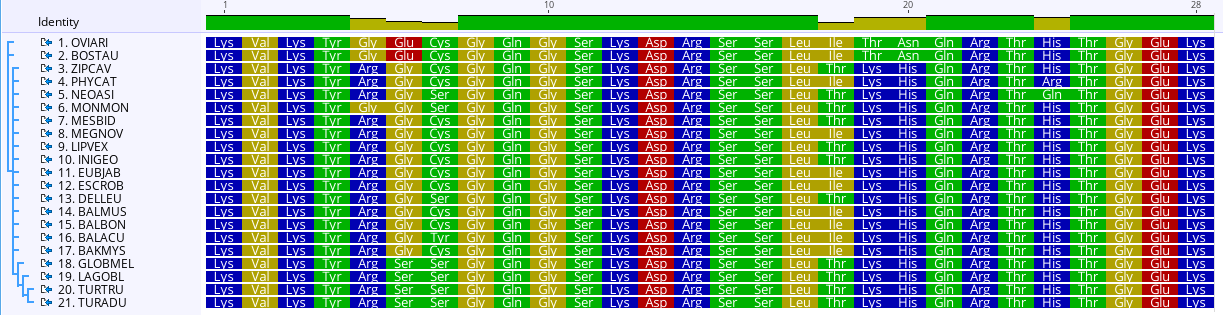

Supplement: Supplementary file 5 — Additional File 5. Alignment of the first ZnF in the ZNF array of Artiodactyla. [file 12864_2022_8305_MOESM5_ESM.png]

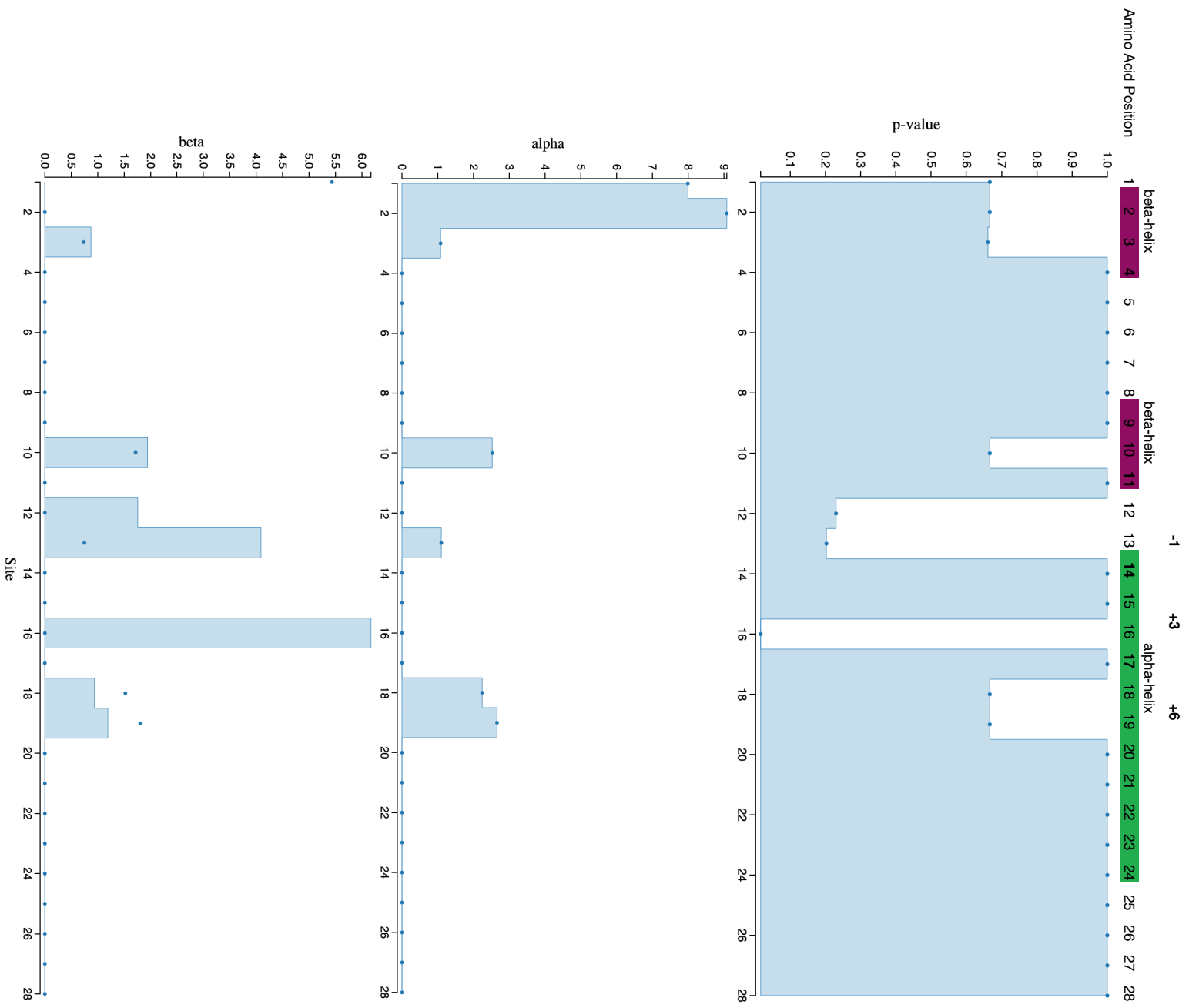

Supplement: Supplementary file 7 — Additional File 7. Signals of selection on amino-acid ZnF identified in this study. [file 12864_2022_8305_MOESM7_ESM.pdf]

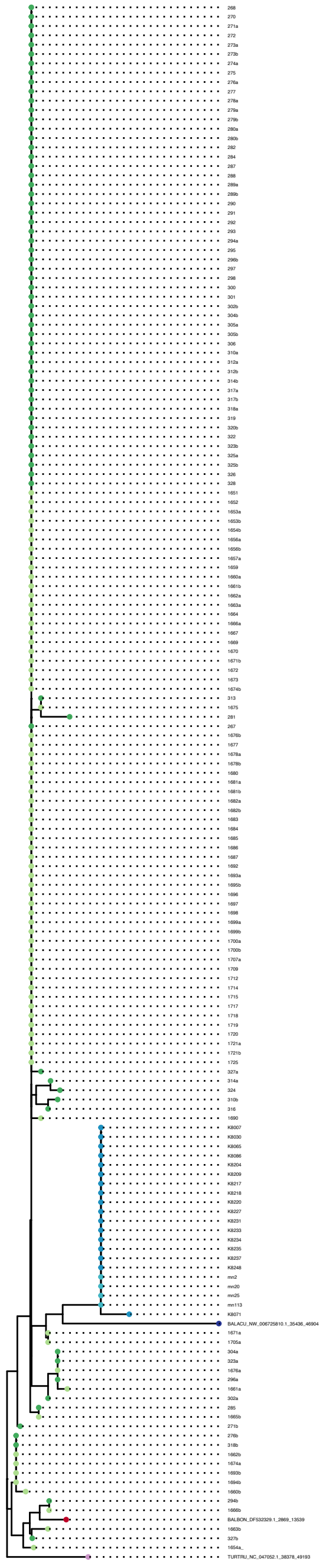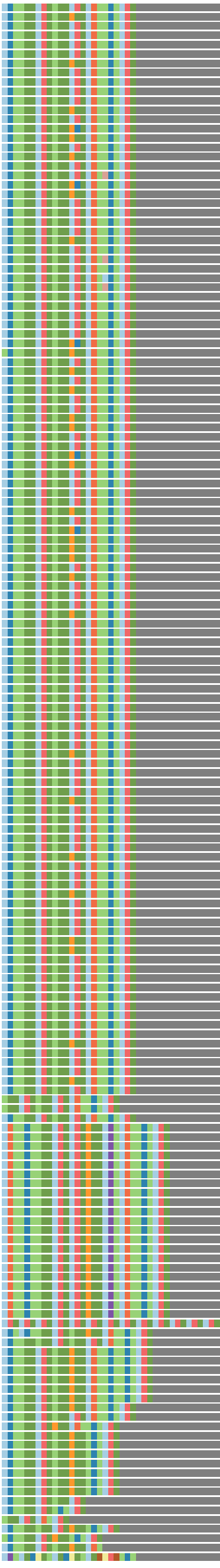

Supplement: Supplementary file 9 — Additional File 9. Prdm9 Phylogenetic analyses including hypervariable sites. [file 12864_2022_8305_MOESM9_ESM.pdf]

**A****K=2**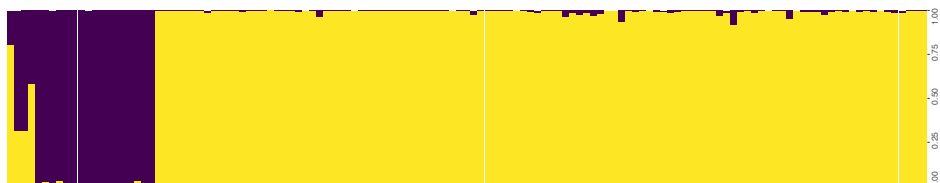**K=3**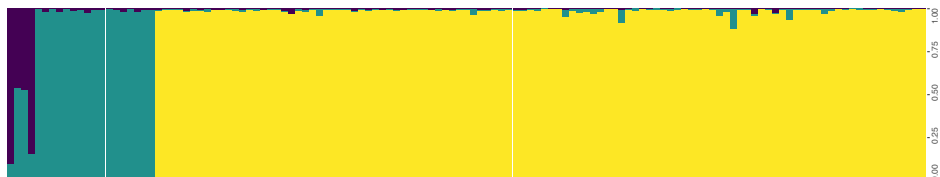**K=4**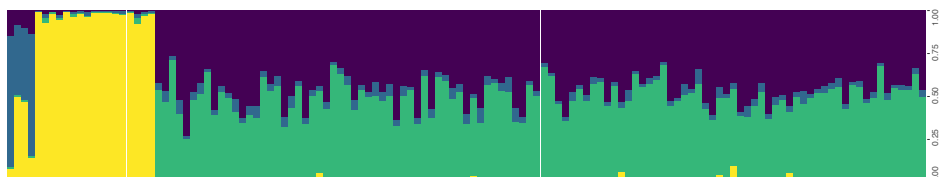**K=5**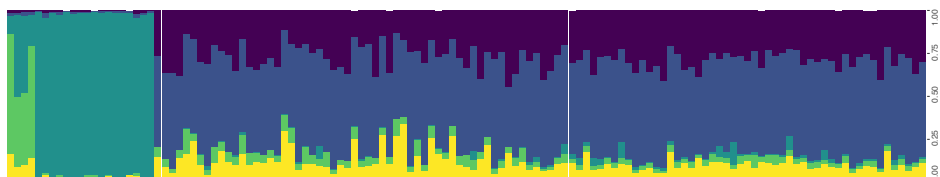**NP****NA****ANIV****ANV****Ancestry****B**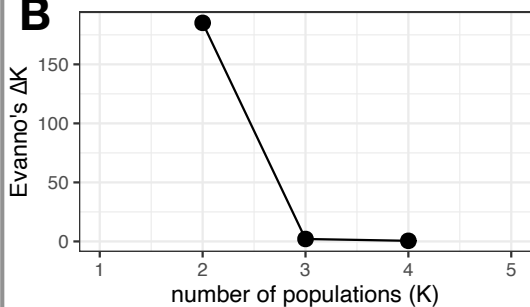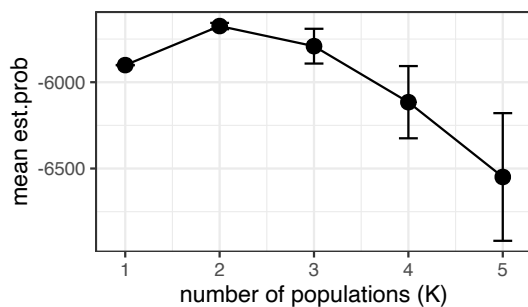

Supplement: Supplementary file 11 — Additional File 11. Population structure analyses on a set of ten hypervariable microsatellite loci. [file 12864_2022_8305_MOESM11_ESM.pdf]

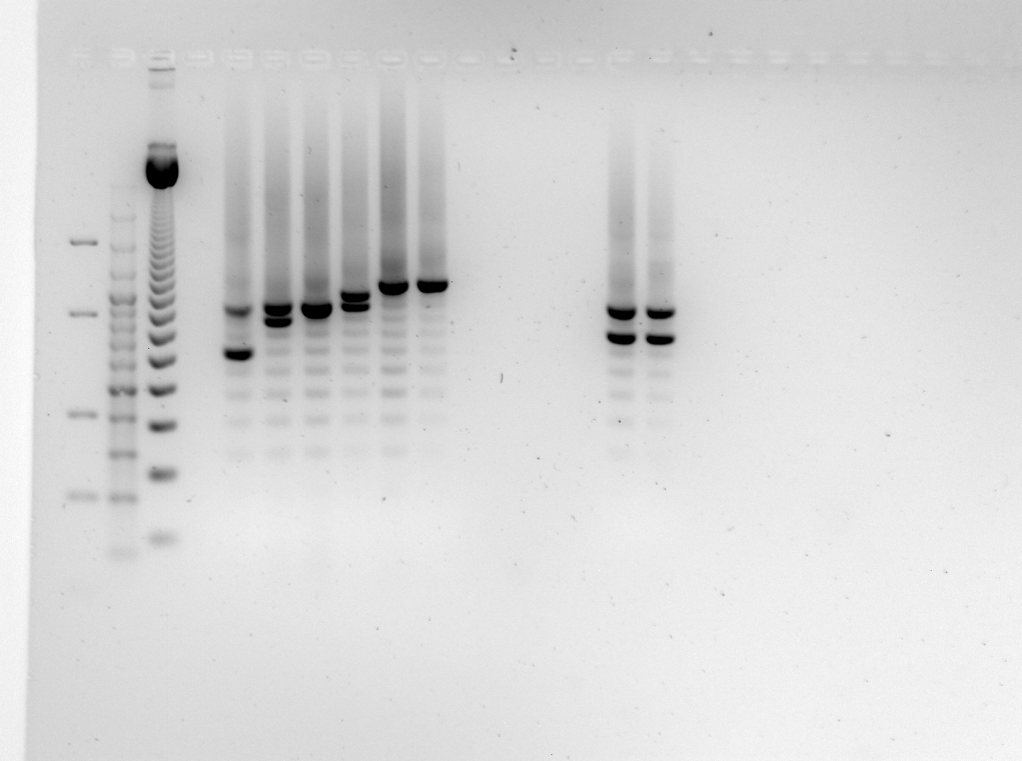

Supplement: Supplementary file 14 — Additional File 14. Uncropped blot of the image used in Fig. 1. [file 12864_2022_8305_MOESM14_ESM.docx]
