# Supplementary material for: Evolution of the recombination regulator PRDM9 in minke whales
Source: BMC Genomics. 2022 Mar 16;23:212. doi: 10.1186/s12864-022-08305-1 (PMC8925151; doi:10.1186/s12864-022-08305-1)
Supplement: Supplementary file 6 — Additional File 6. All PRDM9 ZnF types in minke whales. [file 12864_2022_8305_MOESM6_ESM.pdf]

beta-helix beta-helix alpha-helix

-1 +3 +6

Amino Acid

| | Code | 1 | 2 | 3 | 4 | 5 | 6 | 7 | 8 | 9 | 10 | 11 | 12 | 13 | 14 | 15 | 16 | 17 | 18 | 19 | 20 | 21 | 22 | 23 | 24 | 25 | 26 | 27 | 28 | HMMER bit score | | |
|---|---|---|---|---|---|---|---|---|---|---|---|---|---|---|---|---|---|---|---|---|---|---|---|---|---|---|---|---|---|---|---|---|
| First ZNF | DSK | K | V | K | Y | R | G | C | G | Q | G | S | K | D | R | S | S | L | I | K | H | Q | R | T | H | T | G | E | K | 11.0 | *Ba/Bb | A |
| Internal ZNF | RGG | P | Y | V | C | G | E | C | G | R | D | F | S | R | K | S | G | L | I | G | H | Q | R | T | H | T | G | E | K | 34.1 | | B |
| Internal ZNF | RSR | P | Y | V | C | G | E | C | G | R | D | F | S | R | K | S | S | L | I | R | H | Q | R | T | H | T | G | E | K | 39.0 | | C |
| Internal ZNF | LA(V)R | P | Y | V | C | G | E | C | G | R | D | F | S | L | K | S | A | L | V | R | H | Q | R | T | H | T | G | E | K | 34.3 | *Bb | D |
| Internal ZNF | LSR | P | Y | V | C | G | E | C | G | R | D | F | S | L | K | S | S | L | I | R | H | Q | R | T | H | T | G | E | K | 35.3 | | E |
| Internal ZNF | FGG | P | Y | V | C | G | E | C | G | R | D | F | S | F | K | S | G | L | I | G | H | Q | R | T | H | T | G | E | K | 30.7 | | F |
| Internal ZNF | LD(V)R | P | Y | V | C | G | E | C | G | R | D | F | S | L | K | S | D | L | V | R | H | Q | R | T | H | T | G | E | K | 34.8 | | G |
| Internal ZNF | FSG | P | Y | V | C | G | E | C | G | R | D | F | S | F | K | S | S | L | I | G | H | Q | R | T | H | T | G | E | K | 31.9 | | H |
| Internal ZNF | HSR | P | Y | V | C | G | E | C | G | R | D | F | S | H | K | S | S | L | I | R | H | Q | R | T | H | T | G | E | K | 37.7 | | J |
| Internal ZNF | LNG | P | Y | V | C | G | E | C | G | R | D | F | S | L | K | S | N | L | I | G | H | Q | R | T | H | T | G | E | K | 32.9 | *Bb | K |
| Internal ZNF | N)(R)LN( | P | Y | V | C | G | E | C | G | R | N | F | R | L | K | S | N | L | I | G | H | Q | R | T | H | T | G | E | K | 33.5 | *Bb | K |
| Internal ZNF | (R)LNG | P | Y | V | C | G | E | C | G | R | D | F | R | L | K | S | N | L | I | G | H | Q | R | T | H | T | G | E | K | 32.6 | *Ba/Bb | K |
| Internal ZNF | (N)LNG | P | Y | V | C | G | E | C | G | R | N | F | S | L | K | S | N | L | I | G | H | Q | R | T | H | T | G | E | K | 33.9 | | K |
| Last ZNF | LNG | P | Y | V | C | G | E | C | G | R | D | F | S | L | K | S | N | L | I | G | H | Q | R | T | H | T | G | E | K | 32.9 | | K |
| Last ZNF | (L)LNG | P | Y | L | C | G | E | C | G | R | D | F | S | L | K | S | N | L | I | G | H | Q | R | T | H | T | G | E | K | 29.2 | | K |
| | RGG | P | Y | V | C | A | E | C | G | R | D | F | S | R | K | S | G | L | I | G | H | Q | R | T | H | T | G | E | K | 33.5 | | B* |
| | RGG | P | Y | V | C | G | Q | C | G | R | D | F | S | R | K | S | G | L | I | G | H | Q | R | T | H | T | G | E | K | 33.5 | | B* |
| | RGG | P | Y | V | C | G | E | C | G | G | D | F | S | R | K | S | G | L | I | G | H | Q | R | T | H | T | G | E | K | 29.2 | | B* |
| | RSR | P | Y | V | C | R | E | C | G | R | D | F | S | R | K | S | S | L | I | R | H | Q | R | T | H | T | G | E | K | 37.8 | | C* |
| | LS(V)R | P | Y | V | C | G | E | C | G | R | D | F | S | L | K | S | S | L | V | R | H | Q | R | T | R | T | G | E | K | 19.3 | | E* |
| | FGG(E) | P | Y | V | C | G | E | C | G | R | D | F | S | F | K | S | G | L | I | G | H | Q | R | T | H | T | G | E | E | 30.7 | | F* |
| | FNG | P | Y | V | C | G | E | C | G | R | D | F | S | F | K | S | N | L | I | G | H | Q | R | T | H | T | G | E | K | 33.4 | | M* |
| | HGG | P | Y | V | C | G | E | C | G | R | D | F | S | H | K | S | G | L | I | G | H | Q | R | T | H | T | G | E | K | 33.4 | | N* |
| | LSG | P | Y | V | C | G | E | C | G | R | D | F | S | L | K | S | S | L | I | G | H | Q | R | T | H | T | G | E | K | 31.5 | | O* |
| | PNG | P | Y | V | C | G | E | C | G | R | D | F | S | P | K | S | N | L | I | G | H | Q | R | T | H | T | G | E | K | 32.1 | | P* |
| | LHG | P | Y | V | C | G | E | C | E | R | D | F | S | L | K | S | H | L | I | G | H | Q | R | T | H | T | G | E | K | 29.5 | | L* |
| | RA(V)R | P | Y | V | C | G | E | C | G | R | D | F | S | R | K | S | A | L | V | R | H | Q | R | T | H | T | G | E | K | 38.2 | | Q* |
| | STOP | P | Y | V | C | R | E | G | E | G | V | I | T | K | K | | | | | | | | | | | | | | | 0 | novel | S |

beta-helix beta-helix alpha-helix

-1 +3 +6
