# Supplementary material for: Evolution of the recombination regulator PRDM9 in minke whales
Source: BMC Genomics. 2022 Mar 16;23:212. doi: 10.1186/s12864-022-08305-1 (PMC8925151; doi:10.1186/s12864-022-08305-1)
Supplement: Supplementary file 8 — Additional File 8. Methodological approach. [file 12864_2022_8305_MOESM8_ESM.pdf]

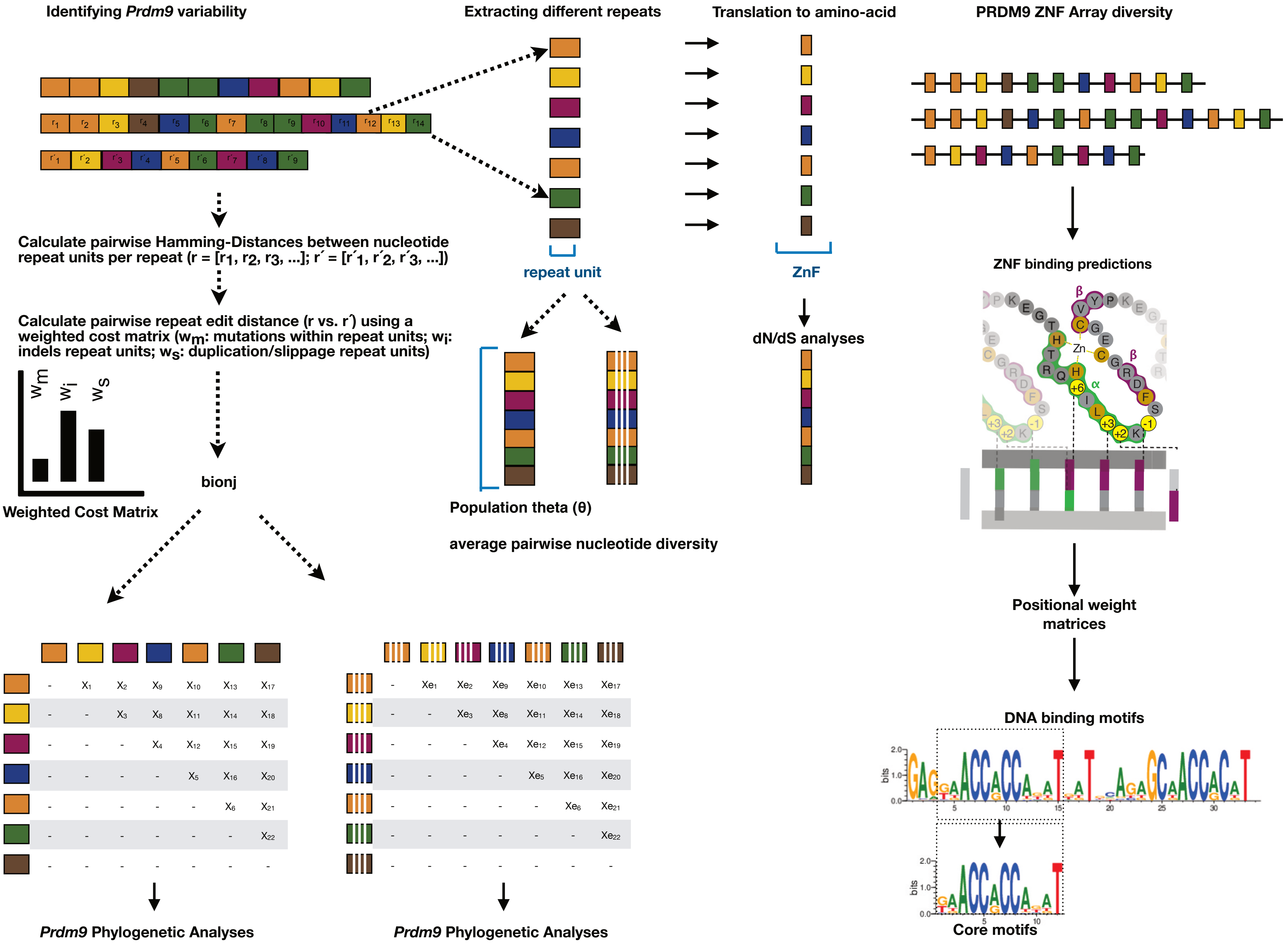
Identifying Prdm9 variability
Extracting different repeats
Translation to amino-acid
PRDM9 ZNF Array diversity
r1 r2 r3 r4 r5 r6 r7 r8 r9 r10 r11 r12 r13 r14
r´1 r´2 r´3 r´4 r´5 r´6 r´7 r´8 r´9
repeat unit
ZnF
Calculate pairwise Hamming-Distances between nucleotide repeat units per repeat (r = [r1, r2, r3, ...]; r´ = [r´1, r´2, r´3, ...])
Calculate pairwise repeat edit distance (r vs. r´) using a weighted cost matrix (wm: mutations within repeat units; wi: indels repeat units; ws: duplication/slippage repeat units)
wm wi ws
Weighted Cost Matrix
bionj
Population theta (θ)
average pairwise nucleotide diversity
dN/dS analyses
ZNF binding predictions
Zn
α
β
Positional weight matrices
DNA binding motifs
bits
Core motifs
X1 X2 X9 X10 X13 X17
X3 X8 X11 X14 X18
X4 X12 X15 X19
X5 X16 X20
X6 X21
X22
Xe1 Xe2 Xe9 Xe10 Xe13 Xe17
Xe3 Xe8 Xe11 Xe14 Xe18
Xe4 Xe12 Xe15 Xe19
Xe5 Xe16 Xe20
Xe6 Xe21
Xe22
Prdm9 Phylogenetic Analyses
Prdm9 Phylogenetic Analyses
