# Supplementary material for: Evolution of the recombination regulator PRDM9 in minke whales
Source: BMC Genomics. 2022 Mar 16;23:212. doi: 10.1186/s12864-022-08305-1 (PMC8925151; doi:10.1186/s12864-022-08305-1)
Supplement: Supplementary file 10 — Additional File 10. Descriptive statistics of microsatellites. [file 12864_2022_8305_MOESM10_ESM.docx]

| Locus | Origin | A | Allele sizes | HE | Ho | FIS | Hs |
| --- | --- | --- | --- | --- | --- | --- | --- |
| GATA028 | AN IV | 20 | 148-272 | 0,93 | 0,74 | 0,21 | 2,73 |
|  | AN V | 20 | 148-274 | 0,92 | 0,88 | 0,05 | 2,67 |
|  | NA | - | - | - | - | - | - |
|  | NP | - | - | - | - | - | - |
|  |  |  |  |  |  |  |  |
| GT575 | AN IV | 26 | 190-262 | 0,95 | 0,95 | 0 | 3,00 |
|  | AN V | 29 | 185-262 | 0,95 | 0,97 | -0,02 | 3,06 |
|  | NA | 8 | 212-240 | 0,85 | 0,89 | -0,06 | 1,89 |
|  | NP | 5 | 206-216 | 0,89 | 1,00 | -0,22 | 1,56 |
|  |  |  |  |  |  |  |  |
| GT310 | AN IV | 18 | 81-135 | 0,88 | 0,78 | 0,11 | 2,41 |
|  | AN V | 18 | 81-137 | 0,90 | 0,82 | 0,09 | 2,46 |
|  | NA | 5 | 113-127 | 0,59 | 0,33 | 0,43 | 1,01 |
|  | NP | 6 | 107-121 | 0,93 | 0,75 | 0,14 | 1,73 |
|  |  |  |  |  |  |  |  |
| EV001 | AN IV | 13 | 103-147 | 0,83 | 0,95 | -0,14 | 1,97 |
|  | AN V | 14 | 105-141 | 0,82 | 0,78 | 0,04 | 1,97 |
|  | NA | 5 | 139-153 | 0,72 | 0,56 | 0,22 | 1,35 |
|  | NP | 4 | 131-151 | 0,86 | 0 | 1,00 | 1,39 |
| GATA098 | AN IV | 9 | 76-108 | 0,83 | 0,89 | -0,08 | 1,85 |
|  | AN V | 10 | 70-108 | 0,84 | 0,97 | -0,16 | 1,94 |
|  | NA | 5 | 80-96 | 0,73 | 0,72 | -0,01 | 1,35 |
|  | NP | 3 | 84-92 | 0,68 | 1,00 | -0,65 | 0,97 |
|  |  |  |  |  |  |  |  |
| GT509 | AN IV | 19 | 179-219 | 0,92 | 0,89 | 0,03 | 2,66 |
|  | AN V | 20 | 179-217 | 0,93 | 0,92 | 0,00 | 2,70 |
|  | NA | 10 | 191-213 | 0,85 | 1,00 | -0,19 | 1,97 |
|  | NP | 5 | 195-211 | 0,89 | 1,00 | -0,22 | 1,56 |
| GATA417 | AN IV | 17 | 188-264 | 0,92 | 0,93 | -0,02 | 2,58 |
|  | AN V | 19 | 184-260 | 0,92 | 0,92 | -0,01 | 2,60 |
|  | NA | 8 | 212-240 | 0,86 | 0,94 | -0,11 | 1,91 |
|  | NP | 5 | 204-242 | 0,89 | 0,75 | 0,11 | 1,56 |
|  |  |  |  |  |  |  |  |
| GT023 | AN IV | 14 | 90-118 | 0,87 | 0,81 | 0,07 | 2,24 |
|  | AN V | 15 | 86-118 | 0,88 | 0,95 | -0,08 | 2,36 |
|  | NA | 8 | 92-108 | 0,77 | 0,78 | -0,02 | 1,64 |
|  | NP | 5 | 94-110 | 0,86 | 1,00 | -0,27 | 1,49 |
|  |  |  |  |  |  |  |  |
| EV037 | AN IV | 18 | 184-222 | 0,91 | 0,49 | 0,46 | 2,53 |
|  | AN V | 16 | 184-222 | 0,93 | 0,52 | 0,44 | 2,59 |
|  | NA | 7 | 192-208 | 0,72 | 0,72 | -0,02 | 1,52 |
|  | NP | 5 | 176-196 | 0,86 | 1,00 | -0,27 | 1,49 |
|  | Origin | A |  | He | Ho | FIS | Hs |
| Average | AN IV | 17,1 |  | 0,89 | 0,82 | 0,07 | 2,44 |
|  | AN V | 17,9 |  | 0,90 | 0,86 | 0,04 | 2,48 |
|  | NA | 7 |  | 0,76 | 0,74 | 0,03 | 1,58 |
|  | NP | 4,75 |  | 0,86 | 0,81 | -0,05 | 1,47 |
