# Supplementary material for: Evolution of the recombination regulator PRDM9 in minke whales
Source: BMC Genomics. 2022 Mar 16;23:212. doi: 10.1186/s12864-022-08305-1 (PMC8925151; doi:10.1186/s12864-022-08305-1)
Supplement: Supplementary file 12 — Additional File 12. Primers used in this study. [file 12864_2022_8305_MOESM12_ESM.docx]

| Method | Name | Sequence | | Source |
| --- | --- | --- | --- | --- |
| Full-length Prdm9 amplification | Prdm9bal_Full_F | | CCA CGG CCA AAG ATG CTT TC | This publication |
| Prdm9 exon 10 | Prdm9bal_ZnFa_F | | GAG CTG AGG TAT GTG GTC GC | This publication |
|  | Prdm9bal_Full + ZnFa_R | | ACT CAT GGG GGC AGG AGT AT | This publication |
| STR genotyping | Ev001_F | | [JOE] CCC TGC TCC CCA TTC TC | [[40](#_ENREF_40)] |
|  | Ev001_R | | ATA AAC TCT AAT ACA CTT CCT CCA AC | [[40](#_ENREF_40)] |
|  | Ev037_F | | [HEX] AGC TTG ATT TGG AAG TCA TGA | [[40](#_ENREF_40)] |
|  | Ev037_R | | TAG TAG AGC CGT GAT AAA GTG C | [[40](#_ENREF_40)] |
|  | Gata028_F | | [6FAM] AAA GAC TGA GAT CTA TAG TTA | [[80](#_ENREF_80)] |
|  | Gata028_R | | CGC TGA TAG ATT AGT CTA GG | [[80](#_ENREF_80)] |
|  | Gata098_F | | [6FAM] TGT ACC CTG GAT GGA TAG ATT | [[80](#_ENREF_80)] |
|  | Gata098_R | | TCA CCT TAT TTT GTC TGT CTG | [[80](#_ENREF_80)] |
|  | Gata417_F | | [JOE] CTG AGA TAG CAG TTA CAT GGG | [[80](#_ENREF_80)] |
|  | Gata417_R | | TCTGCTCAGGAAATTTTCAAG | [[80](#_ENREF_80)] |
|  | Gt023_F | | [HEX] GTT CCC AGG CTC TGCACT CTG | [[81](#_ENREF_81)] |
|  | Gt023_R | | CATTTCCTACCCACCTGTCAT | [[81](#_ENREF_81)] |
|  | Gt211_F | | [HEX] CTG CTC TAT TCT ATG AAA GCA | [[81](#_ENREF_81)] |
|  | Gt211_R | | CTCCAGTATACCTATCTTGTC | [[81](#_ENREF_81)] |
|  | Gt310_F | | [6FAM] GAA TAC TCC CAG TAG TTT CTC | [[81](#_ENREF_81)] |
|  | Gt310_R | | TAA CTT GTG GAA GAT GCC AAC | [[81](#_ENREF_81)] |
|  | Gt509_F | | [6FAM] CAG CTG CAA AAC CTT GAC ATT | [[81](#_ENREF_81)] |
|  | Gt509_R | | GTA AAA TGT TTC CAG TGC ATC | [[81](#_ENREF_81)] |
|  | Gt575_F | | [HEX] TAT AAG TGA ATA CAA AGA CCC | [[81](#_ENREF_81)] |
|  | Gt575_R | | ACC ATC AAC TGG AAG TCT TTC | [[81](#_ENREF_81)] |
| Sex-determination | ZFX_R | | CAC TTA TGG GGG TAG TCC TTT | [[82](#_ENREF_82)] |
|  | ZFY_R | | ATT ACA TGT CGT TTC AAA TCA | [[82](#_ENREF_82)] |
|  | ZFYX_F | | [6FAM] ATA GGT CTG CAG ACT CTT CTA | [[82](#_ENREF_82)] |
| Mitochondrial D-loop sequencing | BP15851(M13F) | | GTAAAACGACGGCCAGTGAAGAAGTATTACACTCCACCAT | [[19](#_ENREF_19)] |
|  | MN312(M13R) | | CAGGAAACAGCTATGACCCGTGATCTAATGGAGCGGCCA | [[19](#_ENREF_19)] |
|  | MT3(M13REV) | | CAGGAAACAGCTATGACCCATCTAGACATTTTCAGTG | [[19](#_ENREF_19)] |
|  | MT4(M13F) | | GTAAAACGACGGCCAGTCCTCCCTAAGACTCAAGGAAG | [[19](#_ENREF_19)] |
