# Supplementary material for: Evolution of the recombination regulator PRDM9 in minke whales
Source: BMC Genomics. 2022 Mar 16;23:212. doi: 10.1186/s12864-022-08305-1 (PMC8925151; doi:10.1186/s12864-022-08305-1)
Supplement: Supplementary file 13 — Additional File 13. Public mitochondrial resources. [file 12864_2022_8305_MOESM13_ESM.docx]

| Species | Common name | Alias | Taxid | Url-sequence |
| --- | --- | --- | --- | --- |
| Balaenoptera acutorostrata | Minke whale | Balacu | 9767 | https://www.ncbi.nlm.nih.gov/nuccore/x72006.1 |
| Balaenoptera acutorostrata | Minke whale | Balacu | 9767 | https://www.ncbi.nlm.nih.gov/nuccore/ay230267.1 |
| Balaenoptera bonaerensis | Antarctic minke whale | Balbon | 33556 | https://www.ncbi.nlm.nih.gov/nuccore/m60408.1 |
| Balaenoptera borealis | Sei whale | Balbor | 9768 | https://www.ncbi.nlm.nih.gov/nuccore/nc_006929.1 |
| Balaenoptera borealis | Sei whale | Balbor | 9768 | https://www.ncbi.nlm.nih.gov/nuccore/ap006470.1 |
| Balaenoptera brydei | Bryde whale | Balbry | 255365 | https://www.ncbi.nlm.nih.gov/nuccore/ap006469.1 |
| Balaenoptera musculus | Blue whale | Balmus | 9771 | https://www.ncbi.nlm.nih.gov/nuccore/nc_001601.1 |
| Balaenoptera omurai | Omura whale | Balomu | 255217 | https://www.ncbi.nlm.nih.gov/nuccore/ab201257.1 |
| Balaenoptera physalus | Fin whale | Balphy | 9770 | https://www.ncbi.nlm.nih.gov/nuccore/nc_001321.1 |
| Balaenoptera physalus | Fin whale | Balphy | 9770 | https://www.ncbi.nlm.nih.gov/nuccore/kc572709.1 |
| Balaena mysticetus | Bowhead whale | Balmys | 27602 | https://www.ncbi.nlm.nih.gov/nuccore/nc_005268.1 |
| Eschrichtius robustus | Grey whale | Escrob | 9764 | https://www.ncbi.nlm.nih.gov/nuccore/nc_005270.1 |
| Eschrichtius robustus | Grey whale | Escrob | 9764 | https://www.ncbi.nlm.nih.gov/nuccore/x72200.1 |
| Eubalaena glacialis | North Atlantic right whale | Eubgla | 27606 | https://www.ncbi.nlm.nih.gov/nuccore/nc_037444.1 |
| Lipotes vexillifer | Yangtze river dolphin | Lipvex | 118797 | https://www.ncbi.nlm.nih.gov/nuccore/nc_007629.1 |
| Megaptera novaeangliae | Humpback whale | Megnov | 9773 | https://www.ncbi.nlm.nih.gov/nuccore/nc_006927.1 |
| Orcinus orca | Killer whale | Orcorc | 9733 | https://www.ncbi.nlm.nih.gov/nuccore/nc_023889.1 |
| Physeter catodon | Sperm whale | Phycat | 9755 | https://www.ncbi.nlm.nih.gov/nuccore/nc_002503.2 |
| Tursiops truncatus | Common bottlenose dolphin | Turtru | 9739 | https://www.ncbi.nlm.nih.gov/nuccore/nc_012059.1 |
